# Coral record of southeast Indian Ocean marine heatwaves with intensified Western Pacific temperature gradient

**DOI:** 10.1038/ncomms9562

**Published:** 2015-10-23

**Authors:** J. Zinke, A. Hoell, J. M. Lough, M. Feng, A. J. Kuret, H. Clarke, V. Ricca, K. Rankenburg, M. T. McCulloch

**Affiliations:** 1 School of Earth and Environment, The University of Western Australia, 35 Stirling Highway, Crawley, Western Australia 6009, Australia; 2 UWA Oceans Institute, 39 Fairway, Nedlands, Western Australia 6009, Australia; 3 Australian Institute of Marine Science, PMB 3, Townsville MC, Queensland 4810, Australia; 4 Department Environment and Agriculture, Curtin University of Technology, Kent Street, Bentley, Western Australia 6102, Australia; 5 School of Geography, Archaeology & Environmental Studies, University of the Witwatersrand, Wits 2050, South Africa; 6 Department of Geography, University of California Santa Barbara, Santa Barbara, California 93106, USA; 7 ARC Centre of Excellence for Coral Reef Studies, James Cook University, Queensland 4811, Australia; 8 Commonwealth Scientific and Industrial Research Organisation (CSIRO), Floreat, Western Australia 6014, Australia; 9 ARC Centre of Excellence for Coral Reef Studies, The University of Western Australia, Crawley, Western Australia 6009, Australia

## Abstract

Increasing intensity of marine heatwaves has caused widespread mass coral bleaching events, threatening the integrity and functional diversity of coral reefs. Here we demonstrate the role of inter-ocean coupling in amplifying thermal stress on reefs in the poorly studied southeast Indian Ocean (SEIO), through a robust 215-year (1795–2010) geochemical coral proxy sea surface temperature (SST) record. We show that marine heatwaves affecting the SEIO are linked to the behaviour of the Western Pacific Warm Pool on decadal to centennial timescales, and are most pronounced when an anomalously strong zonal SST gradient between the western and central Pacific co-occurs with strong La Niña's. This SST gradient forces large-scale changes in heat flux that exacerbate SEIO heatwaves. Better understanding of the zonal SST gradient in the Western Pacific is expected to improve projections of the frequency of extreme SEIO heatwaves and their ecological impacts on the important coral reef ecosystems off Western Australia.

## Abstract (web summary)

Intense marine heatwaves have caused extensive coral bleaching in the Southeast Indian Ocean in recent years, yet their cause remains uncertain. Here, based on a 215-year coral sea surface temperature record, the authors shed light on the mechanistic underpinnings of these extreme events.

## Graphical abstract

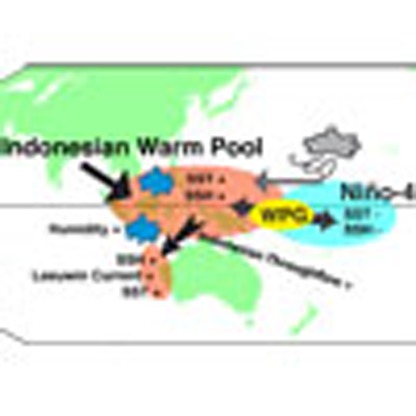

The southeast Indian Ocean (SEIO) region that extends from the western shelf of the Australian continent marks the western boundary of the Indo-Pacific warm pool. It contains several uniquely biodiverse coastal fringing and offshore oceanic and atoll coral reefs (Fig. 1a). These reefs are strongly influenced by warm, poleward-flowing ocean boundary currents, the Holloway and Leeuwin Currents^1^,^2^,^3^. Historically, large-scale warming events resulting in coral bleaching have been relatively limited in the SEIO^3^,^4^,^5^,^6^,^7^, and occurred only locally in response to extreme austral summer sea surface temperatures (SST) during the 1998 El Niño, the most widespread coral bleaching event yet recorded across the Indo-Pacific^6^,^7^. Consequently, the low levels of human development, infrequent coral bleaching, and high recovery potential of disturbed reefs^6^,^7^ suggest that this region may act as a coral refugia during climate warming. However, an extreme La Niña during the austral summer of 2010/11 produced record-high SST leading to the first ever recorded large-scale coral bleaching along 12^o^ of latitude of SEIO reefs^3^,^4^,^5^. This heat wave and similar historical events since 1960 have been dubbed the ‘Ningaloo Niño' and are thought to have been fuelled by an increased Indonesian Throughflow from the Pacific to the SEIO in response to the extreme La Niña, warm Western Pacific SST, high sea-level, a strengthened Leeuwin Current and regional air-sea exchanges^3^,^8^,^9^,^10^. The large-scale drivers of individual heatwaves/‘Ningaloo Niño' that can lead to coral bleaching are still under investigation^3^,^8^,^9^.

Hoell and Funk^11^ and Hoell *et al.*^12^,^13^ showed that during both El Niño and La Niña events the global impacts, including the SEIO, in terms of SST, atmospheric circulation and precipitation, were more severe when the SST anomalies in the western Pacific were strongly opposing those in the central Pacific SST than when the western Pacific SST anomalies were near neutral. Hoell and Funk^11^ described this gradient between the western and central Pacific as the Western Pacific Gradient (WPG)^11^. The WPG^11^ is defined as the standardized difference between the central Pacific (Niño4 region^14^; 5° S–5° N, 160–210° E) and western Pacific SST (0–10° N, 130–150° E) between 1854 to 2010 from the 2° × 2° gridded extended reconstructed SST from NOAA version 3b^15^ (hereafter ERSST^13^; Fig. 1; Supplementary Fig. 1). These studies^11^,^12^,^13^ show that the SST gradient between the central (Niño4 region) and the western Pacific was an important measure of interannual to multi-decadal Pacific climate variability in addition to any previously derived metric of El Niño-Southern Oscillation^14^ (ENSO) or combination of ENSO metrics (see Table 2 of Hoell and Funk^11^).

Due to the remoteness of SEIO reefs and sparseness of historical data, it is hard to quantify the magnitude of temperature stress during past ‘Ningaloo Niño', La Niña and WPG events. It is also not well known whether SEIO heatwaves always have a close association with ENSO on decadal or centennial timescales and whether they preferentially occur during a La Niña-like mean state in the Pacific.

Here we aim to explore historical SST variability and occurrence of warm events in the SEIO and, for the first time, the role of the recently identified SST gradient between the Western and Central Pacific, the WPG^11^, as a large-scale driver in exacerbating heatwaves in the SEIO. To overcome the limitations of short-term instrumental observations, coral proxy records of SST were developed from ten long *Porites* spp. coral cores at three locations covering 11° of latitude (17–28° S) off the coast of Western Australia. Here we present a well-replicated 215-year reconstruction of annual SST for the SEIO based on coral Sr/Ca ratios and stable isotopes. The emergent long-term SST trends and interannual to multi-decadal variability highlight the key role of an increased WPG^11^,^12^,^13^, often in concert with strong La Niña's, in triggering extreme warm events in the SEIO. We trace the cause of historical heatwaves to large energy flux changes associated with an increased WPG^11^ as a result of the western Pacific warming faster than the central Pacific, illustrated by the second rotated empirical orthogonal function of tropical Indo-Pacific SST^11^ (Fig. 1b,c; Supplementary Fig. 1). We show that the magnitude of the WPG varies independently between individual El Niño and La Niña events^8^,^9^,^10^,^11^,^12^,^13^ resulting in modification of the large-scale tropical circulation and precipitation fields^11^,^13^ affecting the energy and heat flux terms, warm water ocean advection through the coastal waveguide and sea-level pressure in the SEIO and ultimately the magnitude of SEIO marine heatwaves.

## Results

### Coral geochemical proxy records of sea surface temperature

**Figure 1:**
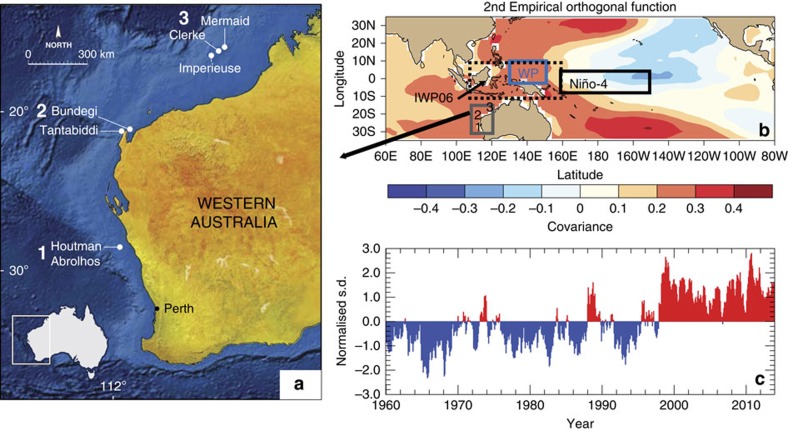
Southeast Indian Ocean reefs and tropical Indo-Pacific SST variability. (**a**) Locations of the three reef areas (1–3) sampled for long coral cores, (**b**) rotated empirical orthogonal function 2 (REOF2) covariance of ERSSTv3b^15^ anomalies, and (**c**) REOF2 time series, 1960–2013, which explains 21% of the variance. The WPG^11^ is defined as the standardized difference between average SST over the Niño4 domain^14^ (black box) and the Western Pacific (WP; blue box), while the Western Australian region is highlighted in grey with coral sampling locations indicated, 1, Houtman Abrolhos, 2, Ningaloo Reef and 3, Rowley Shoals. The black-dashed box marks the Indonesian warm pool region (IWP06 (ref. 26)).

**Figure 2:**
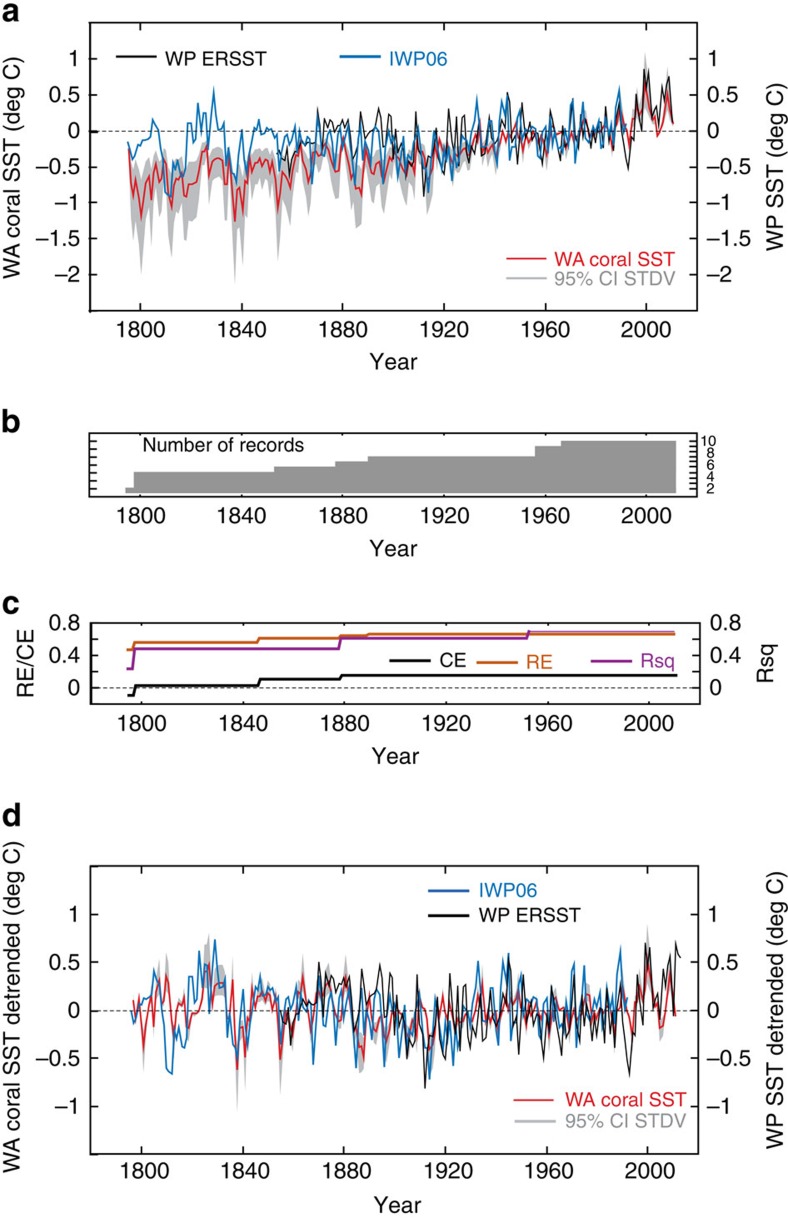
Southeast Indian Ocean coral SST anomaly reconstruction and Western Pacific SST anomalies. (**a**) Reconstructed annual WA coral SST anomaly (red) with 95% confidence interval (grey shaded) based on the spread of both coral and ERSST standard deviations between 1961 and 1990 compared with Indonesian warm pool^26^ (IWP06; blue) and WP SST anomaly reconstructions^15^ (WP ERSST; black). SST anomalies are relative to 1961–1990 mean, (**b**) Number of coral cores through time, (**c**) Reconstruction skill statistics for WA coral SST against regional ERSST^15^ (17–28° S, 113–119° E) are calculated over the validation period (1920–1949) for each proxy nest, including the coefficient of determination (Rsq, magenta), the reduction of error (RE, orange), and the coefficient of efficiency (CE, black) and (**d**) Same as **a**, but detrended time series.

*Porites* coral cores were obtained from the Rowley Shoals (17° S, 119° E), Ningaloo Reef (21° S, 113° E) and the Houtman-Abrolhos Islands (HAI; 28° S, 114° E) between 2008 and 2010 (ref. 16; Fig. 1a). Coral *δ*^18^O and Sr/Ca are widely used as robust proxies for SST^17^,^18^,^19^. While Sr/Ca is considered to be primarily controlled by SST, coral *δ*^18^O can be influenced by both SST and *δ*^18^O_seawater_ through precipitation minus evaporation (P−E) influencing surface ocean salinity^19^. However, interannual changes in P−E at the Rowley Shoals, Ningaloo Reef and HAI are too small to noticeably affect coral *δ*^18^O. Therefore, the *δ*^18^O record primarily reflects variations in SST. We combine two previously published bimonthly-resolved coral *δ*^18^O records^20^,^21^ and two annually resolved Sr/Ca records from HAI^8^ with six new annually resolved Sr/Ca and *δ*^18^O records to reconstruct a robust and well-replicated record of past SST variability in the SEIO (Fig. 2; Supplementary Figs 1–4).

Coral proxy records of SST from three representative reefs of the SEIO were obtained for the period 1795–2010 (Fig. 2; Supplementary Figs 1–2). Least squares linear regressions between individual coral SST reconstructions from the three representative reefs of the SEIO were significant and positive over the record length, typically sharing between 35 to 41% of variance (Supplementary Tables 1–4). The longest records from HAI and Rowley Shoals shared 45% variance between 1798 and 1850 on decadal timescales (>7 years), although the year-to-year variability was not always in phase. Subsequently, a composite annual mean SST record for the SEIO including the three reefs was created by (i) normalizing (subtract mean and divide by standard deviation) each individual coral record to its variance using the time period 1961–1990 shared by all cores, (ii) averaging all records to form a composite chronology for the three reef areas, and (iii) averaging all three composite reef records to form a SEIO composite chronology. We converted our normalized SEIO coral composite proxy record to SST (hereafter WA coral SST) by scaling it to the standard deviation (over the period 1961–1990) of the 2° × 2° gridded ERSSTv3b^15^ and ground-truthed with the 1° × 1° gridded HadISST^22^ (1870–2010) from the UK Hadley Centre (Supplementary Tables 1–7) for the SEIO region 17–28° S, 113–119° E (Fig. 2; Supplementary Figs 3–5).

Least squares linear regression between WA coral SST and the modern SST reconstructions shows significant positive relationships that typically accounted for 20–40% of the SST variance over the entire record length (Supplementary Tables 1–7). Validation statistics show skill of the WA coral reconstruction for most of the record (Fig. 2c). The WA coral SST composite shows long-term warming (Fig. 2a; Supplementary Fig. 3) with strong multi-decadal variability superimposed before 1900 and after 1990. However, our WA coral SST record indicates overall cooler mean SST during the nineteenth century up to the mid-twentieth century than any of the long-term instrumental SST data for the west Australian shelf (Fig. 2; Supplementary Figs 3 and 4). Although there are weaker long-term relationships with ship-based SST reconstructions, the multicore approach provides confidence in the proxy record that would be lacking if based on a single coral core^18^,^19^. The excellent agreement between the coral core composite pre-1900 implies that SST coverage in SST reconstruction data sets based on ICOADS^23^ ship-of-opportunity (Supplementary Figs 3–5) suffers from poor sampling and interpolation from increasingly distant data points.

To assess if mean annual SST capture the major warm events in the SEIO during Ningaloo Niño event years that are normally phase-locked to austral summer and autumn, we evaluated the occurrence of warm anomalies in mean annual versus monthly time scale ERSSTv.3b (ref. 15) since 1950 (Supplementary Table 8). This confirmed that mean annual SST did capture the vast majority (18 out of 26 events) of Ningaloo Niño/Niña event years defined in refs 3, 9 (Supplementary Table 8). Our detrended mean annual WA coral SST is also significantly correlated (*r*=0.59; *P*=0.0001; DF=59) with the Ningaloo Niño index^24^,^25^ between 1948 and 2010, defined for January-February averages (Supplementary Table 9).

### The role of the Western Pacific warm pool for SEIO SST

To assess the role of the Indo-Pacific warm pool on SEIO SST, we compared our WA coral SST reconstruction with SST proxy records from the Indonesian warm pool (detrended) derived from a multi-proxy reconstruction^26^ (hereafter IWP06) and the western Pacific from instrumental data^15^ (hereafter WP SST; Supplementary Table 5). IWP06 extends from 1782 to 1992 and is a composite from annual tree ring and coral records for the Indonesian Archipelago (Fig. 2). For the period 1795 to 1992, the IWP06 and WA coral SST were significantly correlated (*r*=0.48, *P*<0.001; DF=195) and this relationship remained statistically significant after detrending (Supplementary Table 5). WA coral SST indicates overall cooler mean SST during the nineteenth century up to the mid-twentieth century than IWP06. This is in agreement with a recent compilation of tropical coral SST reconstructions^27^ that also showed cooler SST in the Indian Ocean than in the Western Pacific for the entire nineteenth and early twentieth century. Both WA coral SST and IWP06 co-vary on multi-decadal timescales with highest amplitudes between 1795 and 1850 and post 1980 (Fig. 2). The higher amplitude variations in WA coral SST between 1795 and 1850 are solely based on HAI and RS data sets (four cores). The coral records agree best on decadal timescales in this time interval, and show less agreement for year to year events. Our reconstruction skill statistics also revealed lowest skill for the period 1800–1840 with the coefficient of efficiency (CE) just above zero (Fig. 2c). Thus, the absolute magnitude of SST anomalies between 1795 and 1850 in our reconstruction should be interpreted with caution.

**Figure 3:**
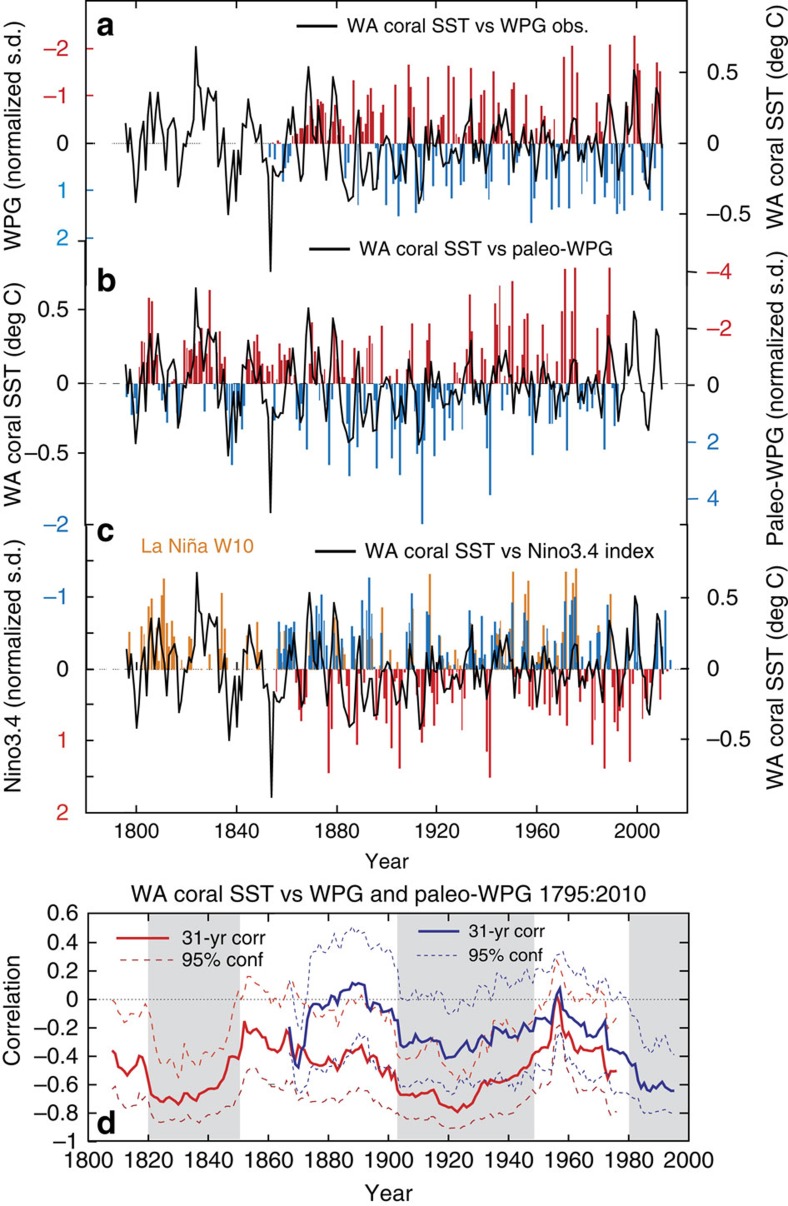
Western Pacific Gradient—SEIO SST relationship. (**a**) WPG from observations (*y* axis inverted) compared to detrended WA coral SST, (**b**) paleo-WPG based on the difference between IWP06 (ref. 26) and Niño3.4 reconstruction^28^, (**c**) Niño3.4 instrumental index (blue= La Niña; red= El Niño) and La Niña-like anomalies in the Niño3.4 reconstruction^28^ superimposed (orange), and (**d**) 31-year running correlations between detrended WA coral SST and paleo-WPG (red solid line, red stippled 95% confidence interval based on a 1,000-sample Monte Carlo simulation^58^) and WPG from observations^11^ (purple solid line, purple stippled 95% confidence interval). Grey shaded areas indicates periods with statistically significant correlations (>95%).

WA coral SST was also significantly correlated with WP SST (r=0.55; *P*<0.001; DF=154), and this relationship was statistically significant after detrending (Supplementary Table 5). The highest correlation between WA coral SST and WP SST was found after 1980 (*r*=0.68; *P*<0.001; DF=28). The same holds for the correlation between WA coral SST and the Niño4 index^14^ (*r*=−0.68; *P*<0.001; DF=28). Post-1980, WA coral SST was also significantly correlated with the WPG^11^ (*r*=−0.69; *P*=0.002; DF=28; Fig. 3a). These results indicate that post 1980, SST in the SEIO was most strongly connected with the western Pacific rather than the Indian Ocean, especially the warm pool and Niño4 region, and the WPG^11^ (Supplementary Tables 5–7; Supplementary Figs 7–9).

### The role of the west pacific gradient for SEIO SST

To assess the importance of western Pacific forcing of SEIO warm events we used the WPG^11^ defined as the standardized difference between the central Pacific (Niño4 region^14^; 5° S–5° N, 160–210°E) and western Pacific SST (0–10° N, 130–150° E) between 1854 and 2010 from ERSST^15^ (Fig. 1). We assessed the long-term stability of the relationship between WA coral SST and the WPG and La Niña events using detrended data (Fig. 3). We also computed a paleo-WPG between 1795 and 1992 from the difference between IWP06 (ref. 26) and a proxy reconstruction of the Niño3.4 index^28^ and extracted La Niña events from both the instrumental Niño3.4 (ref. 14) index and a paleoreconstruction^28^ (Fig. 3). The paleo-WPG reconstruction was shown to be insensitive to the choice of independent Nino3.4 reconstructions^28^,^29^ (detrended) based on paleoclimate data (Supplementary Fig. 10).

WA coral SST (detrended) showed frequent positive anomalies between 1795 and 1900 with highest magnitudes between 1800 and 1850 (Fig. 3a,b). Thus, the co-occurrence of a strongly negative WPG and La Niña appeared to drive warm SST anomalies in the SEIO during the nineteenth century, although set against a cooler mean state of the tropical oceans (Fig. 1; Table 1). Post-1980, we observed an increase in positive SEIO SST anomalies. The majority of warm years over the entire record length correspond to past La Niña events^28^,^29^ of varying strength and a moderate to strong negative WPG (Fig. 3a-d; Table 1). From Table 1 we can conclude that 51 out of 58 years with positive WA coral SST anomalies occurred during strong or moderate negative WPG years (WPG index is negative indicating a warm Western Pacific and cool central Pacific).

To assess the multi-decadal relationship between WA coral SST, ENSO and the WPG we computed 31-year running correlations (Fig. 3e; Supplementary Figs 7–9). We found the highest correlation with the observed WPG and Niño-4 after 1980 at levels unprecedented since at least 1854 (Supplementary Figs 7–9). However, the correlations with the paleo-WPG indicate significantly higher correlations than with the observed WPG throughout the record, being highest between 1800 to 1850, 1900 to 1950 and post 1980. Both observed and paleo-WPG agree on the strengthening relationship with WA coral SST since 1980 (Fig. 3e). However, the paleoreconstruction reveals that the recent strong relationship is not unprecedented and mostly likely part of natural multi-decadal oscillations in the WPG. In contrast, the correlation between WA coral SST and WA ERSST with the Niño-4 (ref. 14) index and a Nino3.4 reconstruction^28^ was stable for most of the twentieth century, yet weaker for most of the nineteenth century (Supplementary Figs 7–9). Thus, we conclude that the paleo-WPG showed stronger connectivity with the SEIO for both the nineteenth and twentieth century than the paleo-Niño3.4 (ref. 14) index. However, the change in ENSO connectivity centred around 1820 could also be related to larger uncertainty in our WA coral SST and/or the paleo-Niño3.4 (ref. 14) index (Supplementary Fig. 9). Niño3.4 (refs 14, 29) paleoclimate reconstructions slightly differ in the early 1800's in the number of La Niña years and that might have affected our analysis.

Nevertheless, the WA coral SST anomalies after 1990 are significantly warmer than any event in the past with 1989, 1996, 1999 and 2008 being the hottest years. The WA coral SST (Fig. 2) identified the hottest year on record in 1999/2000, which corresponds to one of the strongest La Niña and the most extreme negative WPG^11^ events up to the year 2010 (Fig. 1). These recent warm anomalies were also exacerbated by the long-term warming trend of the WP^11^ and the SEIO^8^,^30^ (Fig. 2a). The years following 2008 have seen continuously high SST in both regions^24^,^30^ marked by a strongly persistent negative WPG^11^ (Fig. 3a) and resulting in several coral bleaching events off WA between 2011 and 2013 (ref. 24).

Sea surface height^31^ (SSH) is a dynamic measure of connectivity between the Western Pacific and the SEIO through oceanic waveguides^3^,^8^,^24^. Our WA coral SST was significantly correlated with SSH in the WP, including the WPG region, between 1958 and 2010 (*r*=0.59; *P*<0.001; DF=45) and from the Indonesian Throughflow region to the southwest Australian coast (Supplementary Fig. 6). The correlation between the WPG and SSH mirrors the results from WA coral SST with even higher correlations (*r*=−0.80; *P*<0.001; DF=45). The connection is largely due to the existence of the equatorial and coastal waveguides along the coast of west Australia^3^ on decadal time scale driven by the WPG^11^ and ENSO^3^,^9^,^24^.

**Figure 4:**
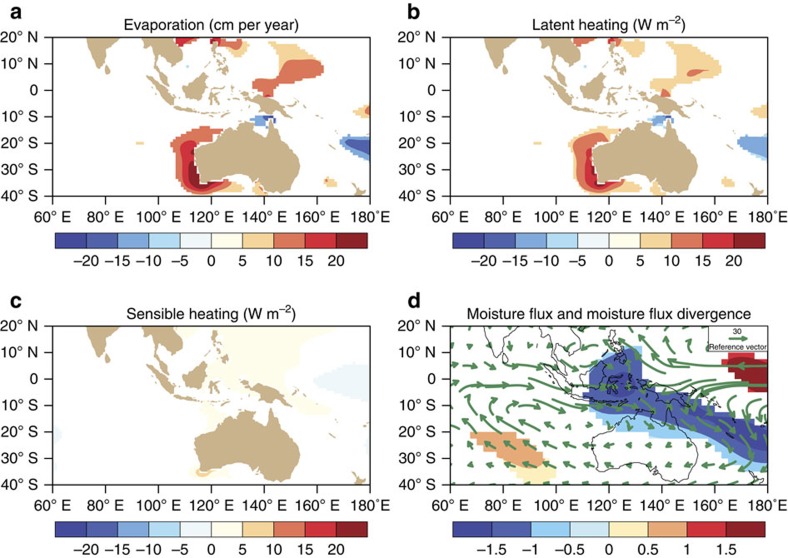
Heat and energy flux terms related to changes in the West Pacific gradient. (**a**) Surface ocean evaporation^32^, (**b**) latent heat flux^32^, (**c**) sensible heat flux^32^ and (**d**) vertically integrated moisture flux (kg m^−1^ s^−1^) and moisture flux divergence (coloured) related to the change in the WPG between 1958 and 2012 (ref. 33). (**a**–**d**) show the flux relationship as composites between negative and positive WPG gradient occurrences. The number of positive and negative occurrences are 8 (16 in total), which correspond to 33% of the 58-year record. Positive flux in **a** to **c** means the ocean is gaining heat. All shadings in (**a**–**d**) are significant to *P*<0.05 using a two-tailed Monte Carlo test.

The question arises how the WPG can force heatwaves in remote coral reefs of the SEIO over large spatial scales. Foremost, the WPG, during and independently from ENSO, remotely influences SEIO warm events by (1) enhancing easterly equatorial wind stress in the western Pacific^11^, (2) inducing equatorial downwelling Rossby waves in the western Pacific which then propagate along the west Australian coast as coastal Kelvin waves^8^,^24^ to the SEIO generating warm SST anomalies (Supplementary Fig. 6) and (3) generating positive SLP anomalies over the Western Pacific that induce negative SLP and cyclonic meridional wind anomalies in the SEIO^3^,^8^,^9^,^10^. Thus, for strong SEIO warm events a strongly negative WPG intensifies the atmospheric and oceanic processes typically associated with La Niña^11^. The opposite holds for El Niño events and positive WPG^11^. We tested our hypothesis that the WPG is also remotely driving changes in the lower troposphere over the northwestern coast of Australia which have been shown to be of paramount importance to enhance the magnitude of SEIO marine heatwaves^3^,^8^,^9^,^10^,^24^. We computed composites for negative and positive WPG from objectively analysed air-sea fluxes (OAFlux)^32^ and NCEP–NCAR Reanalysis I (ref. 33) for the global oceans available from 1958 to 2012. Figure 4 shows that the atmospheric circulation during strong negative WPG (and La Niña) is moistening the lower troposphere in the western part of the Indonesian warm pool and the northwest Australian shelf, which reduces the latent heat flux from the surface (Fig. 4b) and reduces the amount of energy drawn from the sea surface thereby causing an increase in SSTs. The opposite is true for strong positive WPG episodes (El Niño events). The vertically integrated moisture flux drawn from NCEP–NCAR Reanalysis I (ref. 33) for 1958 to 2012 indicates a cyclonic circulation in the Indian Ocean off the west coast of Australia (Fig. 4d), which increases the flux of moisture into the region (one effect of this is enhanced rainfall^9^). The cyclonic atmospheric circulation and low SLP were found to be one of the key drivers of the 2011 marine heat wave^3^,^9^. Furthermore, there are significant reductions in evaporation and latent heat flux (Fig. 4a,b), which cause increases in SST accompanied by very small changes in the sensible heat flux (Fig. 4c). These changes in heat and energy flux terms are also associated with reduced wind speeds as suggested in Marshall *et al.*^25^.

## Discussion

Our results imply that over the past 215 years the WPG^11^ was a key player in Indo-Pacific climate connectivity in addition to ENSO-driven SST anomalies. Recent changes in the WPG, combined with strong WP warming after the Indo-Pacific climate regime shift of the late 1990s (refs 24, 34), are driving significant thermal anomalies impacting coral reef ecosystems over several thousands of kilometres from the Indonesian seas to the southern coast of Western Australia and along the southwest Pacific. The abrupt rise in western Pacific SST in the late 1990s was also addressed by recent studies^8^,^11^,^24^,^35^,^36^. Hoell and Funk^11^ showed that the abrupt warming of the west Pacific has resulted in a more negative WPG, which in turn has forced strong drought-inducing teleconnections across the Northern Hemisphere and the circum-Indian Ocean. However, the period since the late 1990s is also characterized by marked oscillations in WP and SEIO SST associated with the Interdecadal Pacific Oscillation coupled with more frequent La Niña and Ningaloo Niño events^3^,^4^,^5^,^6^,^7^,^8^,^9^,^10^,^24^. The increased magnitude of thermal stress anomalies in the SEIO since the late 1990s revealed by our WA coral SST and similar events in the SW Pacific are supported by recent work on the 2011–2013 Ningaloo Niño's^3^,^8^,^9^,^24^,^25^, by the severity of warming following the 2011 La Niña along the Western Australian coast^3^,^4^,^5^,^6^,^7^,^8^,^9^,^10^,^24^ and observed mass coral bleaching in Papua New Guinea and the southwest Pacific Islands (Fiji, Solomon)^37^ during the 1999/2000 protracted La Niña. These findings point to an uncertain future for WP and SEIO coral reef ecosystems, despite their not being exposed to many of the local pressures degrading other reefs around the world (for example, pollution, overfishing), because they are living close to their upper thermal threshold.

The temporal evolution of the WPG with future ocean warming combined with decadal climate variability discussed here will determine the thermal stress level that coral reefs in the teleconnected regions experience. The western pole of the WPG index has been warming strongly in the past two decades and, together with Indian Ocean SST, closely tracks radiative anthropogenic forcing^38^,^39^. This western Pacific warming is at the heart of the recent strengthening of the Pacific Walker Circulation that ultimately strengthened the climate connectivity between the WP and the SEIO presented here^35^,^36^,^38^. Our results also reveal that, at times, the WP and central Pacific warm or cool at similar rates, resulting in small changes to the WPG. Consequently, during such periods of low WPG variability we find weaker relationships with our SEIO SST reconstruction. For these periods, ENSO forcing from the Niño4 or Niño3.4 regions will dominate over that of the WP. At times, for instance between 1800 and 1850, both the Niño3.4 index and the WPG showed large amplitude variations on multi-decadal timescales that are mirrored by WA coral SST. Similar large amplitude variations in the early 19th century were also observed in coral records from the Western Pacific Warm Pool^27^,^40^,^41^. This multi-decadal variability in the nineteenth century was most probably related to internal variability of the climate system and appears to be a prominent signal across the Indo-Pacific warm pool. Part of the multi-decadal ups and downs is related to large volcanic eruptions in the period 1800–1850 (ref. 42). Thus, the independent variability of the WPG from ENSO is of pivotal importance for Indo-Pacific climate connectivity and their impacts on the environment and human society. Recently, Cai *et al.*^43^ showed, for a subset of CMIP5 models, a high likelihood for a more intense WPG between the Maritime continent and the central Pacific and intensified La Niña events in twenty-first century climate projections, further highlighting the importance of the coral records presented here. Our work provides an historical perspective on marine heatwaves in the SEIO and shows that recent thermal stress events are likely the result of strong anthropogenic warming that made it easier for natural climate variability to exceed the critical threshold for mass coral bleaching to occur in this previously thought coral refugia region. Improved SST reconstructions and sustained long-term monitoring are keys to our ability to predict the ecological consequences of continued warming for the unique WP and SEIO coral reef socioecological systems.

## Methods

### Core locations and sampling

The Rowley Shoals are located in the eastern tropical Indian Ocean, on the edge of the northwest Australian shelf, forming an extended shelf region of tertiary carbonate composition bounded by shelf edge atolls^44^. Two cores were obtained in 2009 from Imperieuse Reef (Fig. 1), the southernmost reef of the Rowley Shoals, and are part of an ongoing study into the climatological history of Australia's coral reefs conducted by AIMS^16^. Each of the 90 mm diameter cores (IMP05A, 17°5196 S, 118°969 E; 8-m water depth, 3.43-m long) and IMP03A (17°5369 S, 118°974 E, 14-m water depth, 3.11-m long) were taken from bommies of *Porites* genus ∼2-km apart. Two 70-mm diameter cores CLE09 (1.5-m long, 10-m water depth) and MER09 (1.5-m long, 17-m water depth) were obtained from Clerke (northernmost atoll) and Mermaid (halfway between Imperieuse and Clerke) reefs during research cruises of AIMS and the University of Western Australia (UWA), respectively, in 2009.

Ningaloo Reef extends over 300 km and includes the only example in the world of an extensive fringing coral reef on the west coast of a continent. Ningaloo Marine Park (NMP) was listed as a World Heritage site in 2013 (ref. 45). Tantabiddi Reef lies in the northwestern section of NMP and forms a narrow lagoon which provides rapid exchange with the open ocean and the Leeuwin Current (LC). Bundegi Reef lies in the northeastern section of the NMP, an area that extends into the shallow Exmouth Gulf. Coral cores (*Porites* spp.) from Tantabiddi (TNT07C; 21°91 ′S,113°97 ′E; 2.5-m water depth, 1.55 m long) and Bundegi (BUN05C; 21°87 ′S,114°17 ′E; 2-m water depth; 1.78-m long) were drilled in October 2008 by AIMS^16^. Both corals sampled were from inner reef environments located within 200–300 m of the shoreline. Growth rates for the corals were determined from the X-ray photographs and were between 1–1.6 cm per year for Tantabiddi and 1.2–1.9  cm per year Bundegi. The published stable isotope record from Tantabiddi Reef extends from 1878 to 1994 (ref. 20). A core of 2.8 m in length from 3-m water depth was recovered in May 1995 (growth rate ranged between 1.04 and 1.36 cm per year).

Coral cores from the Houtman Abrolhos Islands (HAI; cores HAB10A and HAB05B) are described in detail in refs 8, 21. The HAI are a group of carbonate platforms lying approximately 50–60 km offshore of the western coast of Western Australia (WA), and is the southernmost true coral reef formation SouthEast Indian Ocean^46^ (SEIO). The HAI lie within the path of the LC, and support an astonishing diversity of corals for such a high latitude reef (28.5° S, 113° E). Given their latitude, the HAI are subject to relatively low seasonal SST variation of ∼4 °C, largely attributable to the LC^47^. The intra-annual variation in salinity of ∼0.4 p.s.u. is also low^47^. However, interannual variability of mean annual HAI SST is high, with La Niña years being significantly warmer (annual mean temperature in 2011 was 1.5 °C above twentieth century average SST) than El Niño years^47^.

The cored colonies were all ≥1.5 m in height and on the leeward side of the reef. Cores were extracted using a hydraulic drill, and the hole was then sealed with a concrete plug to prevent microbial infection, colonization of the bore hole and to allow recolonization by the living tissue layer. The use of replicate cores from the same area allowed smoothing of any inconsistencies and minimizing the presence of false signals from localized environmental factors^48^.

Following initial sectioning and preparation by AIMS and UWA, core slices were cut into sections ∼500-mm long using a Buehler IsoMet 1000 precision sectioning saw. Joints between subsections were cut on an interlocking angle to ensure appropriate sampling overlap, thus preserving the chronology. Slices were visually inspected aided by densitometry measurements^16^ for diagenesis along the growth axis, which may impart an artificial cooling/warming signal^48^,^49^ The segments were then cut as needed to allow router access to the principal growth axis and exposing a ledge for milling. Using a Zenbot CNC controlled Hitachi router with a 4-mm routing bit, the ledge was milled 2.5 mm inwards from the growth axis for each consecutive high and low-density band comprising one coral growth year. This excess material was removed to ensure that any surface contaminants from handling could not contaminate the next sample.

Each slab was then cleaned with a reagent-grade solution of sodium hypochlorite (NaOCl) and milli-Q water at a 1:1 ratio for 24 h. This process removes excess organic material, particularly in the tissue layer, whilst preserving the trace element composition of the sample^50^ Excess NaOCl and particulate matter were then removed from the slab by ultrasonic cleaning in deionized water for thirty minutes, with the water replaced at 10-min intervals. Finally, the sections were dried in a Contherm Thermotec 2000 drying oven.

Skeletal density banding was prominent in all cores (visible in X-rays) and were the dominant control for determining age relationships and the orientations of the growth axes given their established use as coral chronometers^51^ (Supplementary Figs 11–16). Luminescence banding was used to confirm the position/orientation of the growth axes where density banding was difficult to interpret in small sections of the cores. Density and luminescence banding on the whole cores showed excellent agreement and thus their combined use enabled the most accurate interpretation of the orientation of the growth axis.

### Sr/Ca and stable isotope analysis

Following the method of Zinke *et al.*^8^, annual samples of ∼50 mg (mean 52.28 mg; s.d.±1.03 mg) of finely powdered core dust were homogenized and then weighed into thoroughly cleaned 5-ml Eppendorf tubes, then dissolved in 2.1 ml of 0.562 N HNO3. The dissolved samples were first diluted to a calcium concentration of 100 p.p.m. by taking an aliquot of 30 μl from the primary dissolution and adding 2.7 ml of 2% HNO3. The final trace element aliquots were prepared at 10 p.p.m. by using a 30 μl aliquot of the first dilution and adding 2.7 ml of 2% HNO3 spiked with trace concentrations of scandium, bismuth, praseodymium and yttrium so that the sensitivity of the instruments could be scaled.

Samples were analysed for trace element concentration on a Thermo Scientific XSERIES 2 quadrupole inductively coupled plasma mass spectrometer. The standard reference material for calibration is the JCp-1 *Porites* sp. standard prepared by the Geological Survey of Japan^52^. All Sr/Ca data are normalized to JCp-1 with Sr/Ca=8.838 mmol mol^−1^. External reproducibility has been checked by repeated analysis (*N*=150) of our in-house coral (*Porites* sp.) standard Davies Reef (DR) which gives Sr/Ca=8.953±0.34% (2*σ*).

The stable oxygen isotope (*δ*^18^O) ratios in Kuhnert *et al.*^20^,^21^ were analysed on a Finnigan MAT 251 mass spectrometer calibrated against NBS-19. They are reported in per mil versus Vienna Pee Dee Belemnite isotope scale (‰ VPDB). Analytical errors of replicate measurements of an internal laboratory standard (Solnhofen limestone) are less than ±0.07 for *δ*^18^O. The *δ*^18^O analyses of the new HAB10A coral record was undertaken at the West Australian Biogeochemistry Centre (WABC) at UWA following the protocol of Paul and Skrzypek^53^. *δ*^18^O was analysed using GasBench II coupled with Delta XL Isotope Ratio Mass Spectrometer (Thermo-Fisher Scientific, Bremen, Germany). All results were expressed using the standard *δ*-notation (*δ*^18^O) and reported in per mil (‰) after normalization to the Vienna Pee Dee Belemnite isotope scale (‰ VPDB). The multi-point normalization was based on three international standards NBS18, NBS19 and L-SVEC, each replicated twice^54^. The analytical uncertainty was lower than ±0.10‰ (1*σ*) for *δ*^18^O. The proxy SST data from this study will be made available through NOAA paleoclimate database ( www.ncdc.noaa.gov/paleo/).

### Age models and reconstructions

We developed all chronologies based on annual density banding assisted by luminescence banding. For the Abrolhos corals, we adopted a second step to assess the agreement between geochemical records where we had additional information based on a long higher resolution sampling (Kuhnert *et al.*^20^). The new cores HAB05B and HAB10A had some horizons where annual banding was not very clear in the X-rays. Therefore, we used the COFECHA tool to cross-date the individual geochemical records with the annualized high-resolution record from Kuhnert *et al.*^20^ (see Methods in Zinke *et al.*^8^). This indicated the critical time intervals in need of optimization in the Abrolhos records. For Ningaloo and Rowley Shoals, no adjustment was needed. Annual band counting for all cores was based on annually sampled records only and all agreed with the number of years in the geochemical records. A composite SST record was created by (i) normalizing (subtract mean and divide by s.d.) each individual coral geochemical record to its variance using the time period 1961–1990 shared by all cores, (ii) averaging all records to form a composite chronology for the three reef areas and (iii) averaging all three composite reef records to form a SEIO composite chronology (hereafter WA coral SST). We converted our normalized SEIO coral composite proxy record to SST by scaling it to the s.d. of ERSSTv3b (ref. 15; over the period 1961–1990), the longest and most reliable continuous SST dataset for the SEIO region, over the region 17–28° S, 113–119° E. To determine the accuracy of the long-term composite record, we calculated the 95% confidence interval in the spread of the SST s.d. (grey shading in Fig. 2a,d) for 1961–1990 in both WA coral SST and ERSSTv3b (ref. 15) and subsequently estimated the spread in the scaling coefficient. We used the maximum spread in the scaling coefficient as uncertainty bounds on our final coral record for both WA coral SST with trend (grey shading in Fig. 2a) and for detrended data (grey shading in Fig. 2d). To allow for the number of coral records decreasing backwards in time, reconstruction skill statistics^55^ for each nest of SST reconstructions starting with 2 records and ending with the composite record of all 10 proxy time series were calculated over the validation period 1920–1949, including the coefficient of determination (Rsq), the reduction of error (RE), and CE (Fig. 2c). Values of RE (CE) above zero indicate some statistical skill in that the reconstructed values over the validation period are better estimates of SST than the mean of the calibration (validation) period. The calibration period was 1950–2008 and comprised two-thirds of the years that the proxy and instrumental SST time series with best data coverage shared in common, with the validation period (1920–1949) comprising the remaining one-third. The choice of the validation period was largely based on the HadSST3 (ref. 56) data sets for the West Australia region (17–28° S, 113-19° E) which showed large data gaps between 1870 and 1920.

### Instrumental data

The reconstructed annual SST was verified against four SST data sets: (1) the NOAA 0.25° × 0.25° gridded Advanced Very High-Resolution Radiometer Optimally Interpolated SST^57^ (AVHRR OISSTv2 1981 to 2010); (2) the 2° × 2° gridded extended reconstructed SST from NOAA version 3b (ref. 15); (3) the 1° × 1° gridded HadISST^22^ (1870–2010); and (4) 5° × 5° gridded HadSST3 (ref. 56), the latter two from the UK Hadley Centre (Supplementary Tables 1–4). We use the Western Pacific SST gradient^11^ (WPG) defined as the standardized difference between the central Pacific (Niño4 region^14^; 5° S–5° N, 160–210° E) and western Pacific SST (0–10° N, 130–150° E) between 1854 and 2010. All data sets were accessed via the Royal Netherlands Meteorological Institute (KNMI) online climate explorer^58^.

### Paleo-WPG from paleoclimate data

We extracted the mean annual Niño3.4 reconstructions from www.ncdc.noaa.gov/paleo/28,29. The Indonesian warm pool SST reconstruction (IWP06 (ref. 26)) was kindly provided by Rosanne D'Arrigo. We extracted La Niña events from two Niño 3.4 paleoreconstructions^28^,^29^. For the Wilson *et al.*^28^ Niño 3.4 reconstruction we selected the negative anomalies that correspond with weak to extreme La Niña events. We computed a paleo-WPG between 1795 and 1992 from the difference between annual mean IWP06 (ref. 23) and a reconstruction of the annual mean Niño 3.4 index^28^. This differs from the observational WPG defined as the standardized difference between the central Pacific (Niño4 region^14^; 5°S–5° N, 160–210°E) and western Pacific SST^15^ (0–10° N, 130–150° E). The IWP06 record does include the western Pacific region (0–10° N, 130–150° E), yet is of larger extent. Currently, no paleoclimatic reconstruction is available for the Niño4 region, so we relied on the Niño3.4 index of Wilson *et al.*^28^ which is significantly correlated with the Niño4 index (*r*=0.82 (*r*=0.76–0.87, 95% confidence interval), *P*<0.001, *N*=138). The Niño3.4 region also represents the central tropical Pacific and shares a large amount of variance with the Niño4 region (*r*=0.92, *P*<0.001, *N*=138) and is therefore considered the best available record to calculate a paleo-WPG. We note that the standard deviation of IWP06 and the Niño3.4 reconstruction are of different magnitude than the observational indices for the WP SST and Niño3.4. Nevertheless, we verified our approach by directly comparing the observed WPG with our paleo-WPG to ensure that the timing and relative magnitudes of anomalies were well matched with the observational record. The correlation between the (detrended) observed WPG and paleo-WPG was significant (*r*=0.67 (*r*=0.57–0.75, 95% confidence interval), *P*<0.001, *N*=138). Considering only data after 1880, where SST observations are considered more reliable, resulted in a higher correlation between WPG and paleo-WPG (*r*=0.77 (*r*=0.69–0.82, 95% confidence interval), *P*<0.001, *N*=112).

## Additional information

**How to cite this article:** Zinke, J. *et al.* Coral record of southeast Indian Ocean marine heatwaves with intensified Western Pacific temperature gradient. *Nat. Commun.* 6:8562 doi: 10.1038/ncomms9562 (2015).

## Supplementary Material

Supplementary Information Supplementary Figures 1-16, Supplementary Tables 1-9 and Supplementary References.

## Figures and Tables

**Table 1:** Occurrence of positive SEIO SST anomalies and the strength of WPG and Nino3.4 events.

	**Strong WPG**	**Moderate WPG**	**Neutral/positive WPG**
Strong La Niña	**1826, 1829**, 1893**, 1910, 1917, 1934, 1943, 1950, 1974, 1989, 1999, 2000, 2008**	**1849, 1863, 1873, 1874**, 1890**, 1894**	1971
Moderate La Niña	**1806, 1870**, 1944, 1945, **1955, 1975, 2001**	1797, **1802, 1823, 1824, 1828, 1835**, 1857**, 1861, 1871, 1880, 1909**, 1939**, 1976, 1996**	**1796**, 1840**, 1876, 1911**, 1984
Weak La Niña	**1805**, 1833, **1879, 1898**, 1933, 1949, **1962**	**1825, 1830, 1832, 1847, 1852, 1872, 1882, 1916, 1932**	**1798**, 1799**, 1809, 1810, 1851**, 1925
Weak El Niño		**1844, 1845, 1846 1850**	

SEIO, southeast Indian Ocean; SST, sea surface temperature; WPG, Western Pacific Gradient

The negative WPG years were grouped into strong (>1 standard deviation (s.d.) of annual mean values) and moderate (<1 s.d.) years, and neutral/positive WPG years (based on the paleo-WPG cross-validated with the instrumental WPG^11^). La Niña years (based on the instrumental data Niño3.4 index^14^ post-1854 and a combination of paleo-Niño3.4 indices pre-1854 (refs 28, 29)) were grouped into strong (>1 s.d.), moderate (>0.5 s.d.) and weak events (<0.5 s.d.) for mean annual values. We also classified weak El Niño years. Years in bold indicate events recorded in the WA coral SST.
