# Supplementary material for: Coral record of southeast Indian Ocean marine heatwaves with intensified Western Pacific temperature gradient
Source: Nat Commun. 2015 Oct 23;6:8562. doi: 10.1038/ncomms9562 (PMC4639796; doi:10.1038/ncomms9562)
Supplement: Supplementary Information — Supplementary Figures 1-16, Supplementary Tables 1-9 and Supplementary References. [file ncomms9562-s1.pdf]

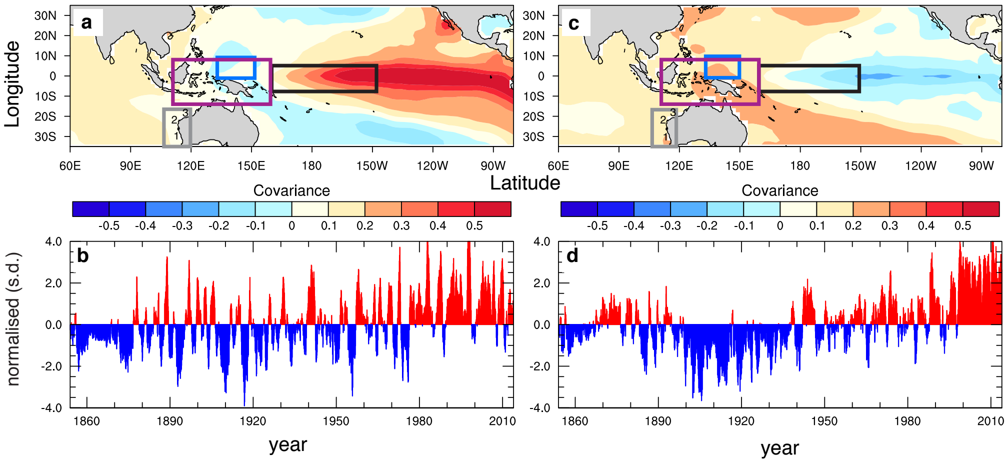

Supplementary Figure 1 – Leading modes of tropical Indo-Pacific SST variability. a) REOF1 and c) REOF2 covariance of ERSSTv3b anomalies[1] (1961-1990) and b) REOF1 and d) REOF2 time series for 1854 to 2013. REOF1 explains 32% and REOF2 21% of the variance, respectively. The WPG[2] is defined as the standardized difference between average SST over the Niño 4 domain[3] (black box) and the WP (blue box), while the WA region is highlighted in grey with coral sampling locations indicated, 1, Houtman Abrolhos, 2, Ningaloo Reef and 3, Rowley Shoals. The magenta box marks the Indonesian warm pool region[24].

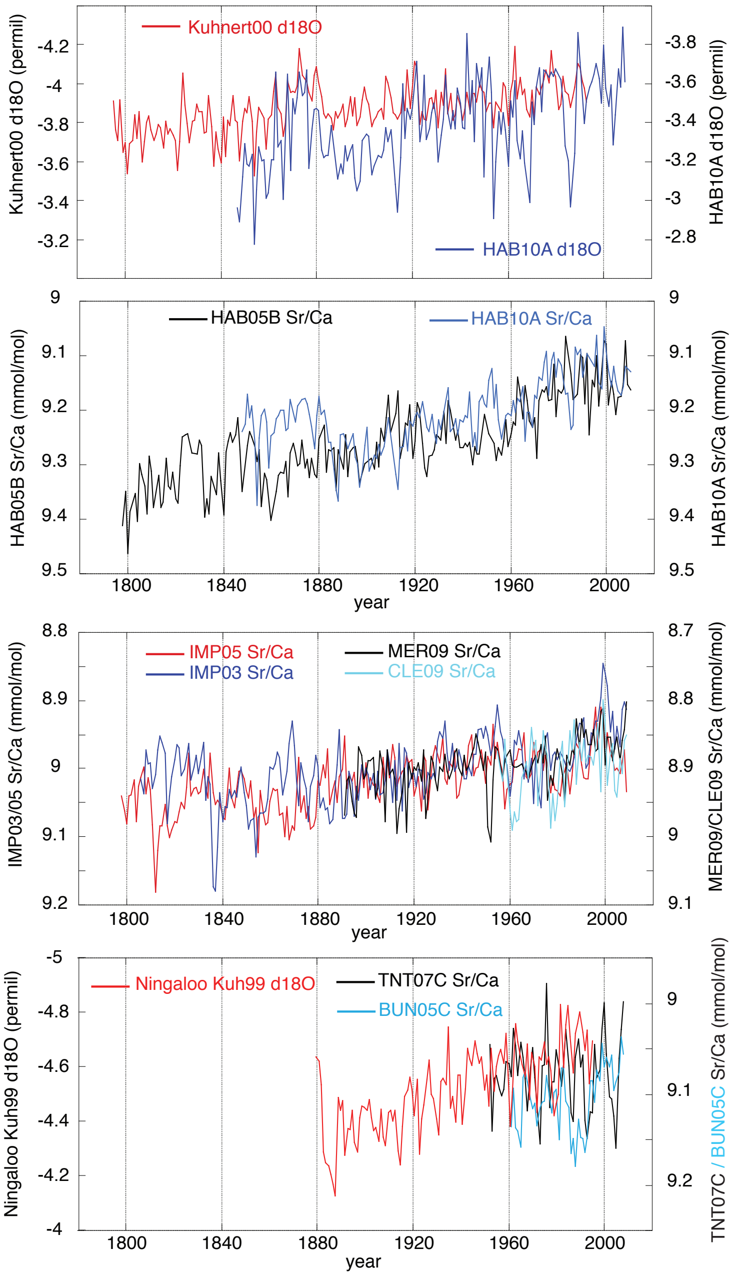

Supplementary Figure 2 – Individual coral mean annual proxy time series for a) Houtman Abrolhos (HAI) oxygen isotopes from Kuhnert et al. 1999[4] and HAB10A, b) Houtman Abrolhos (HAI) Sr/Ca from cores HAB10A and HAB05B, c) Rowley Shoals Sr/Ca from cores IMP03A, IMP05A, MER09 and CLE09, and d) Ningaloo Reef (NING) oxygen isotopes from Kuhnert et al., 2000[5] and new cores TNT7C and BUN05C. Note that Y-axis has been reverted to show negative proxy values that correspond with warmer SST and vice versa for more positive proxy values.

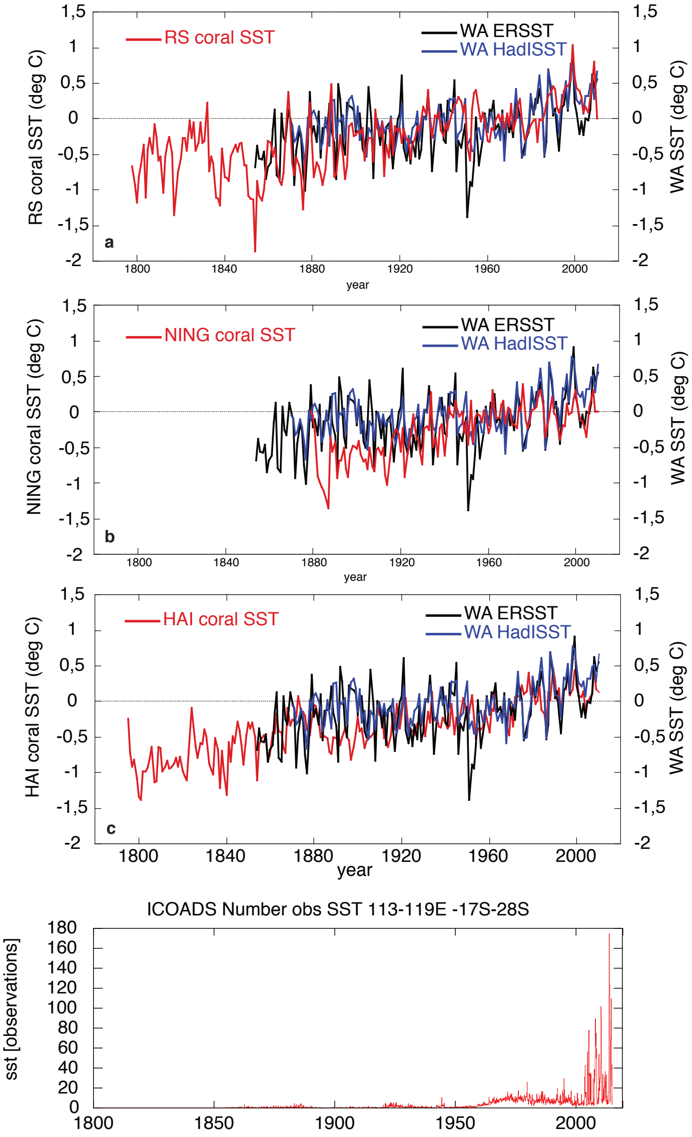

Supplementary Figure 3 – ERSST[1] (black) and HadISST[6] (blue) for the WA region 17-28°S, 113-119°E compared to a) Rowley Shoals (RS), b) Ningaloo Reef (NING), and c) Houtman Abrolhos (HAI) coral SST reconstructions (red). Coral time series shown in geographical order from North to South. Note that the Rowley Shoals time series (in a) agrees best with ERSST (black) and HadISST (blue) for the WA region 17-28°S, 113-119°E over the complete record length. See individual correlations in Supplementary Tables 1 to 7. Lower Panel) Number of SST observations in the ICOADS[7] data base used to reconstruct ERSST and HadISST[6].

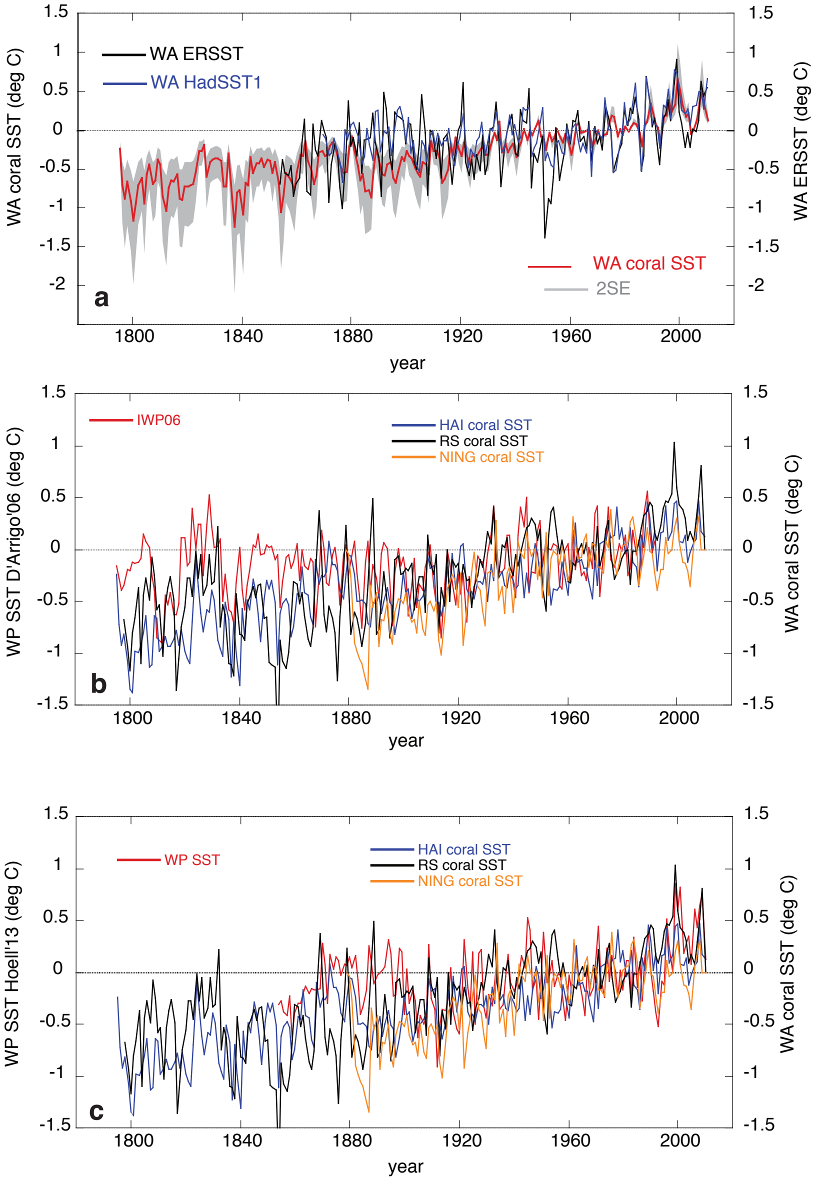

Supplementary Figure 4 – a) Time series of reconstructed annual WA coral SST (red with 2SE grey shaded) anomalies and regional SST from ERSSTv3b[1] (black) and HadISST[6] (blue), b) Rowley Shoals (RS), Ningaloo Reef (NING) and Houtman Abrolhos (HAI) SST reconstructions compared to IWP06[8] (red) after D'Arrigo et al.[8] and c) same as b), but with WP SST (red) after Hoell and Funk[2]. Note the excellent agreement of multi-decadal oscillations and cooler background SST anomalies between 1798 to 1850 in both Rowley Shoals and HAI corals (in b and c) despite being 11° of latitude apart.

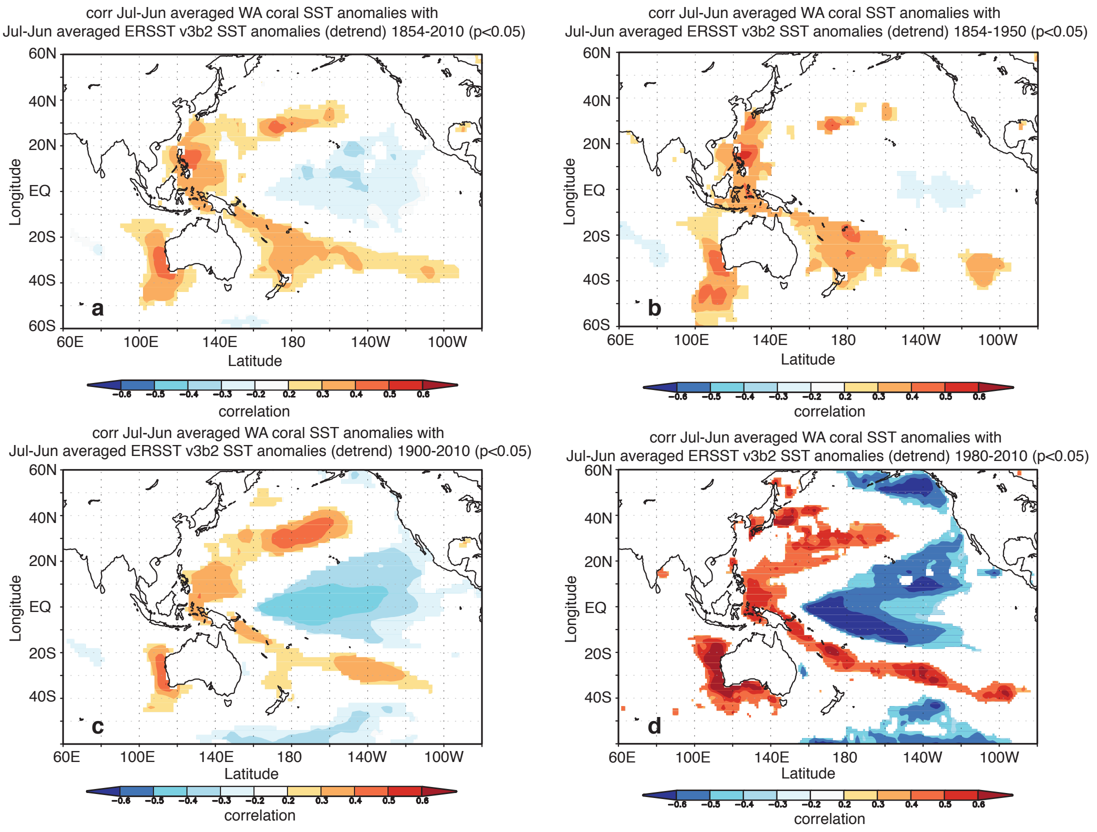

Supplementary Figure 5 – Spatial correlation of WA coral SST anomalies with ERSST[1] anomalies for subperiods. a) 1854 to 2010, b) 1854 to 1950, c) 1900 to 2010, and d) 1981 to 2010. Only correlations significant at the 5% level are shown, computed at KNMI climate explorer[9]. Note the typical ENSO-horseshoe Pacific SST pattern, strong SST gradient between western Pacific and the central Pacific and the stable correlation with southeastern Indian Ocean and southwest Pacific ERSST for all subperiods.

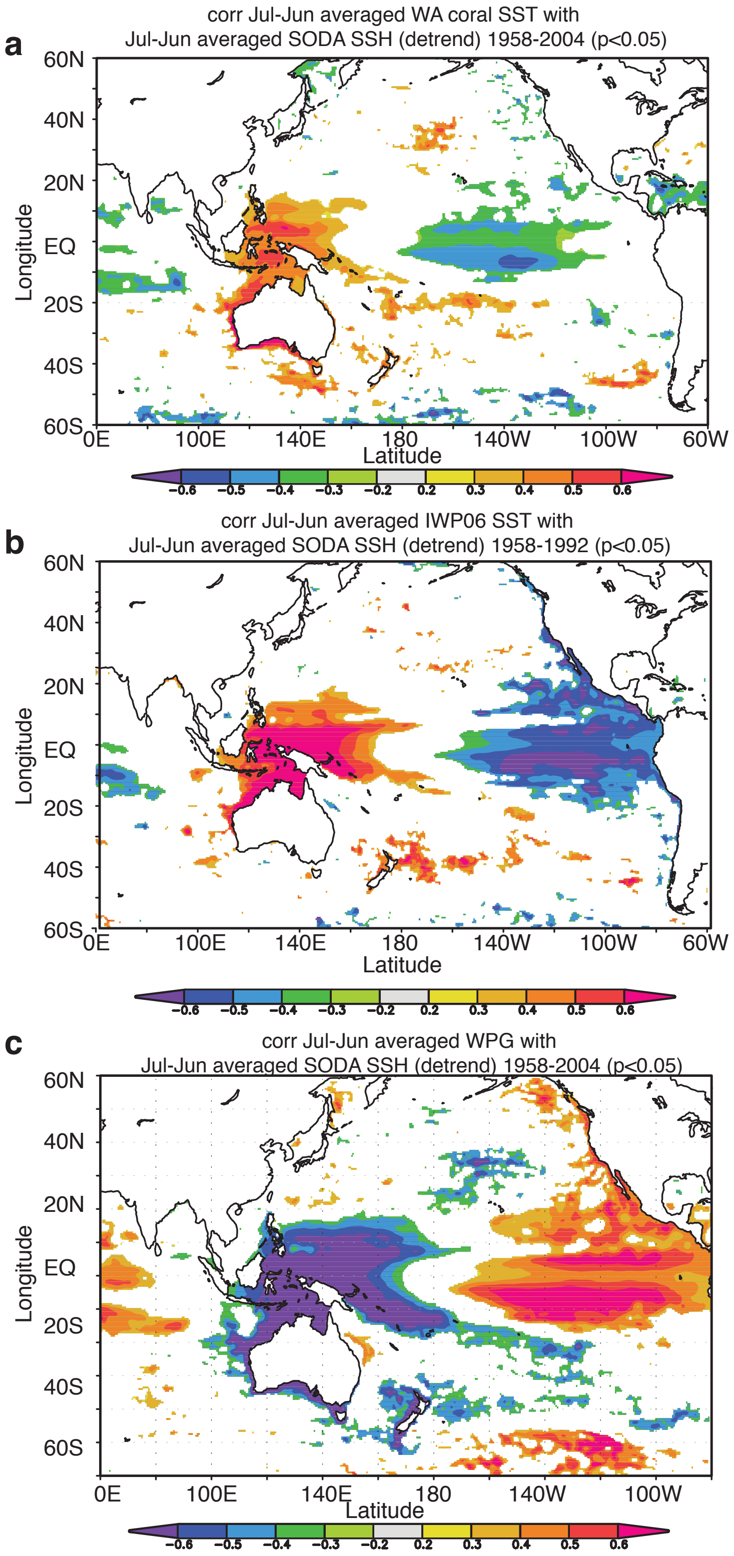

Supplementary Figure 6 – Spatial correlation of SSH from SODA reanalysis[10], 1958-2004 with a) WA coral SST, b) IWP SST from D'Arrigo et al.[8], and c) WPG[2]. Only correlations significant at 5% level are shown, computed at KNMI climate explorer[9].

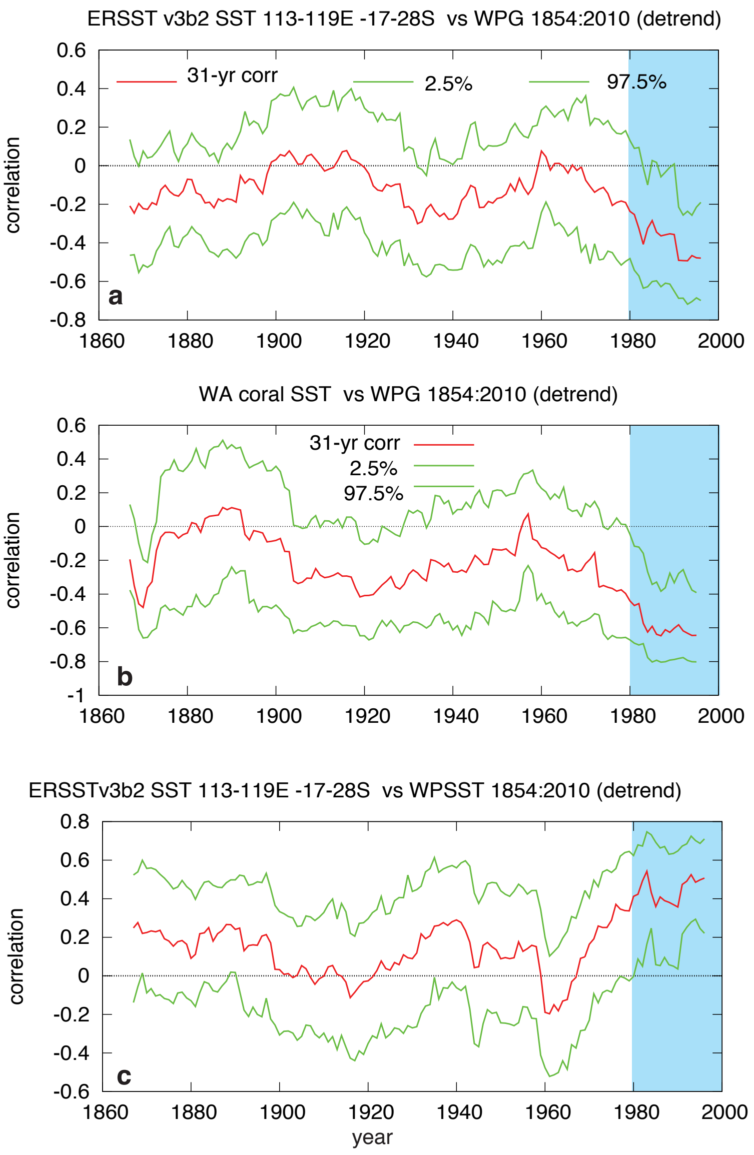

Supplementary Figure 7 – 31-year running correlations (red lines) between detrended a) WA ERSST[1] and WPG[2], b) WA coral SST and WPG[2], and c) WA ERSST and WP SST[1]. The 95% confidence interval is indicated with green solid lines (based on a 1000 sample Monte Carlo simulation). The blue shaded area indicates the period after 1980 with highest correlations and statistically significant (>95%). Correlations computed at http://climexp.knmi.nl/[9].

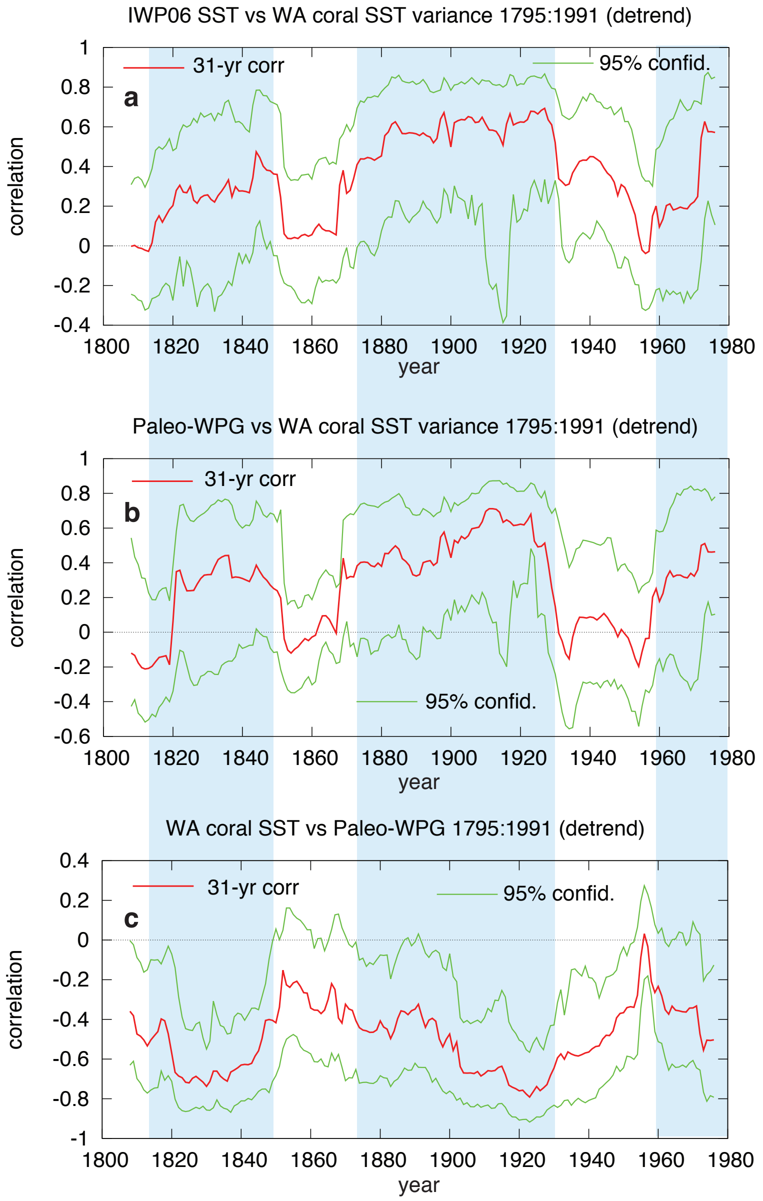

Supplementary Figure 8 – 31-year running variance correlations (red lines) between detrended a) IWP06 SST[8] and WA coral SST, b) WA coral SST and Paleo-WPG compared to c) 31-year running correlation between WA coral SST and Paleo-WPG. The 95% confidence interval is indicated with green solid lines (based on a 1000 sample Monte Carlo simulation). The blue shaded area indicates the period with highest and statistically significant (>95%) correlations in a-c. Correlations computed at http://climexp.knmi.nl/[9]. Our results suggest that the variance in WA coral SST increases when the Western Pacific is warm, its variability enhanced and when the WPG is strong.

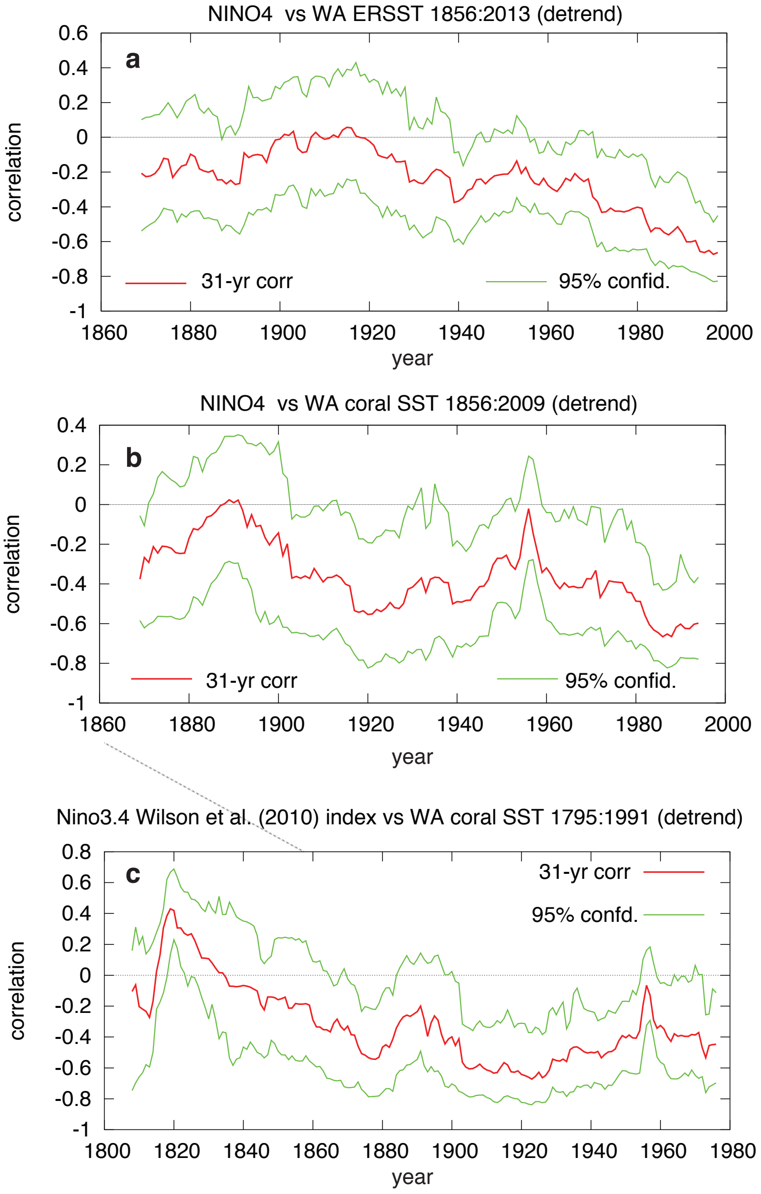

Supplementary Figure 9 – 31-year running correlations (red lines) between detrended a) WA ERSST[1] and Nino4 index[3], b) WA coral SST and Nino4 index[3] and c) Nino3.4 index of Wilson et al. (2010)[11] with WA coral SST. The 95% confidence interval is indicated with green solid lines (based on a 1000 sample Monte Carlo simulation). Correlations computed at http://climexp.knmi.nl/[9].

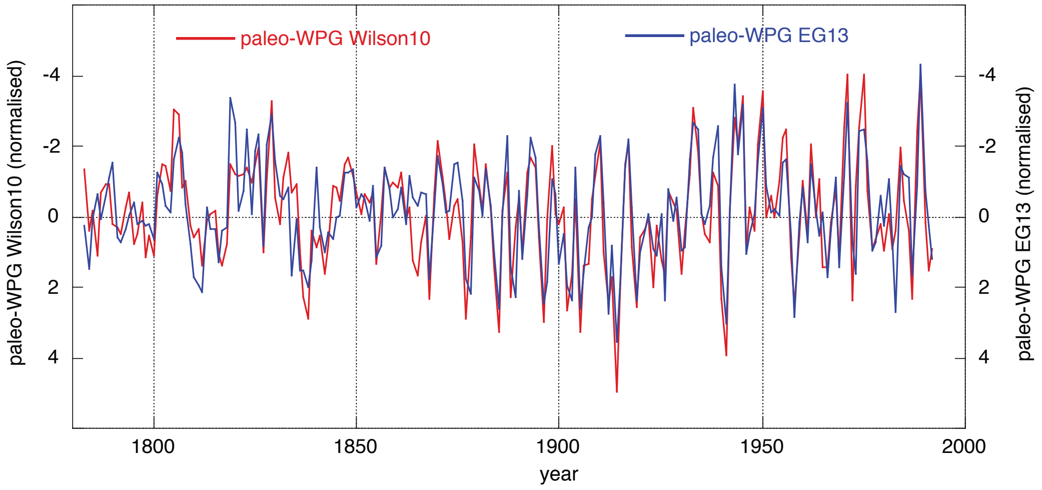

Supplementary Figure 10 – Paleo-WPG reconstructions based on the Nino3.4 index of Wilson et al. 2010[1] (red) and Emile-Geay et al. 2013[12] (blue). Note that both reconstructions strongly co-vary. We used the Wilson et al.[11] (Wilson 10) index because it uses only records from the central and eastern Pacific. The Emile-Geay et al.[12] (EG13) record includes teleconnected regions which we wanted to exclude when computing the paleo-WPG. We therefore decided to use the Wilson index instead of Emile-Geay to compute the paleo-WPG. Nevertheless, we have tested how well the paleo-WPG agrees when using the Emile-Geay record and found a high correlation with the Wilson-based paleo-WPG of r^2=0.68 ($p<0.001$; DF= 208) for the period 1782-1992. The major events are well matched and above all the strong multi-decadal signal in the paleo-WPG between 1800-1850 could be reproduced with Emile-Geay et al.[12].

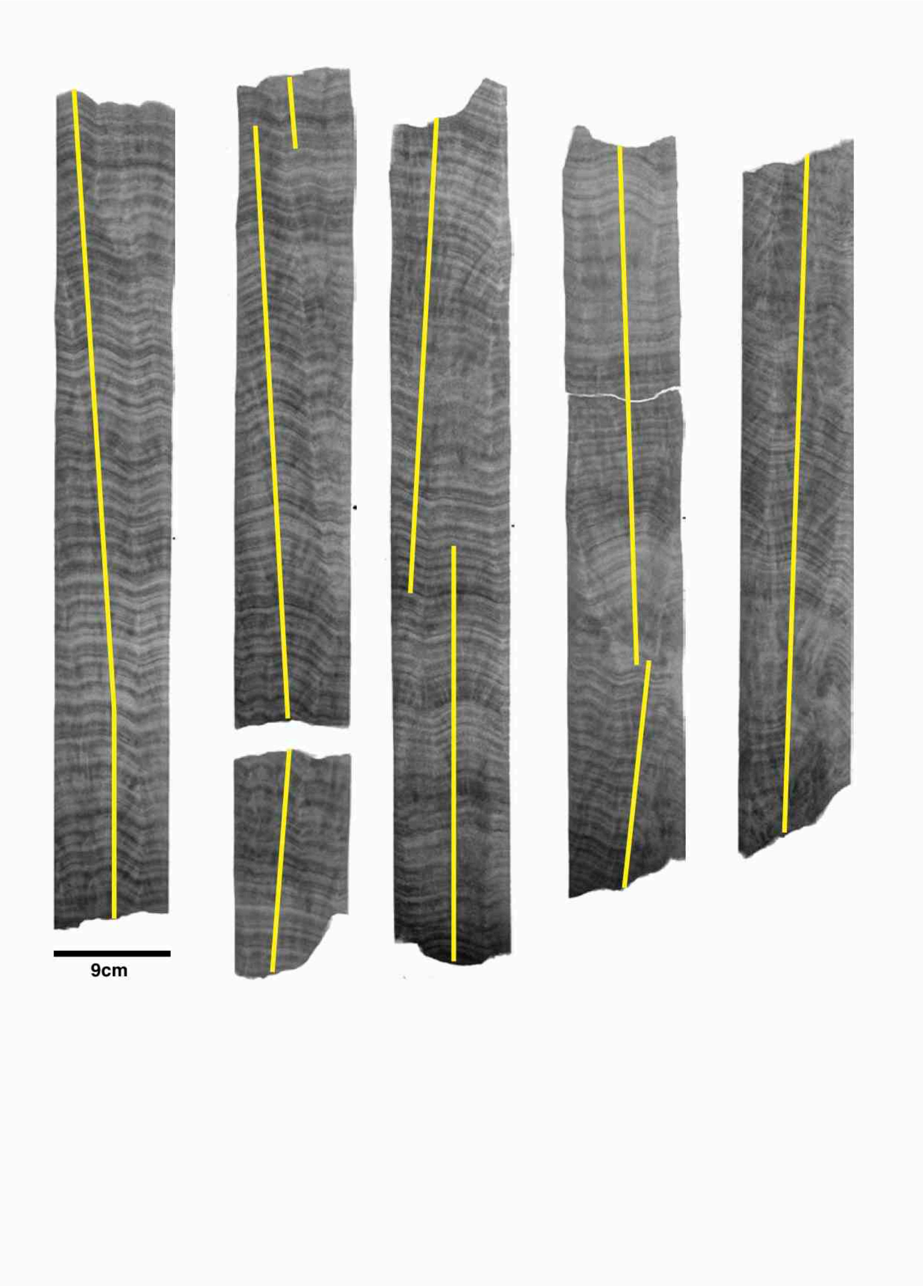

Supplementary Figure 11 – X-ray positive prints of coral core slabs from the Rowley Shoals core IMP05A (Imperieuse Reef) with sampling transects indicated (yellow line).

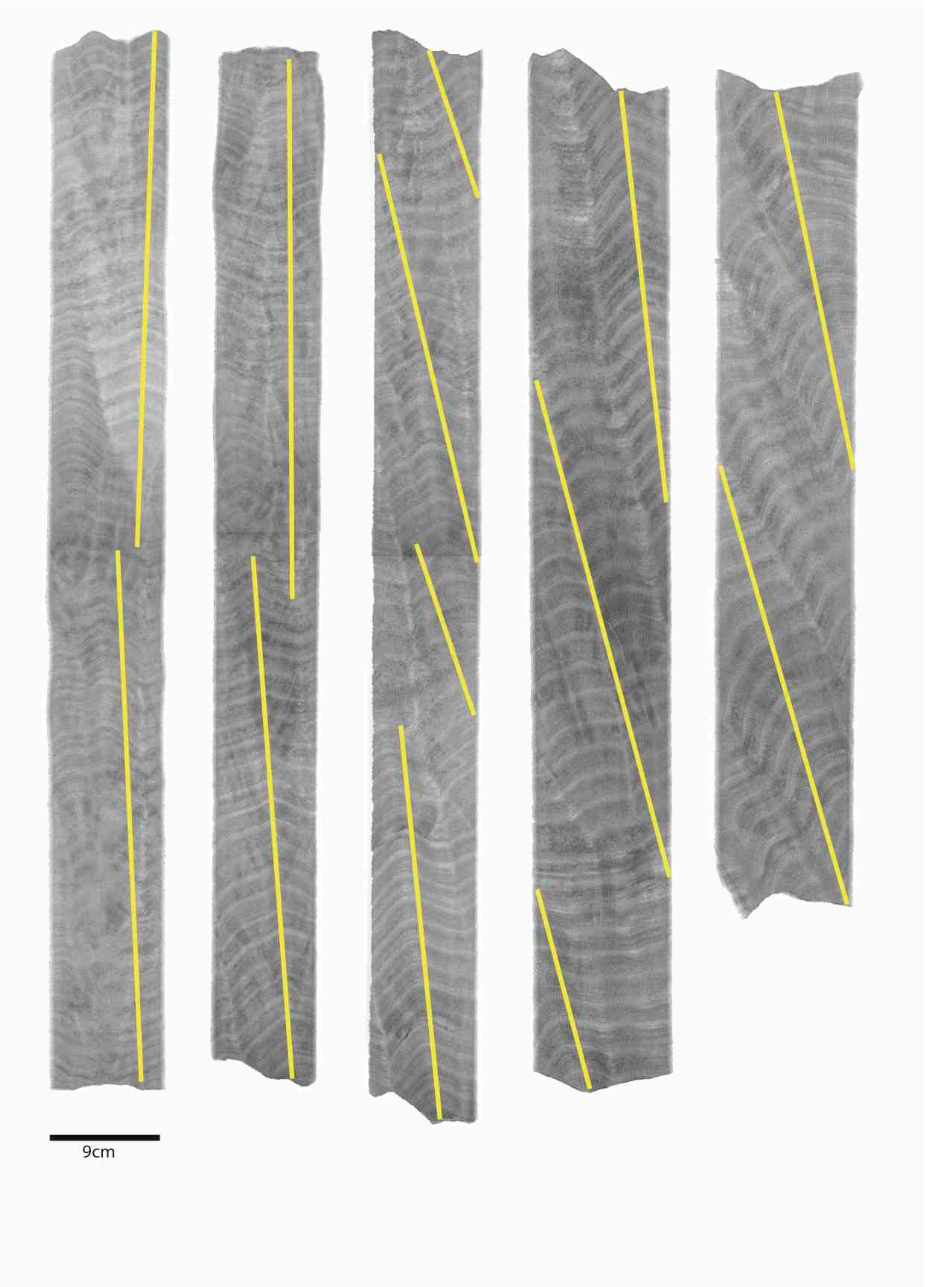

Supplementary Figure 12 – X-ray positive prints of coral core slabs from the Rowley Shoals core IMP03A (Imperieuse Reef) with sampling transects indicated (yellow line).

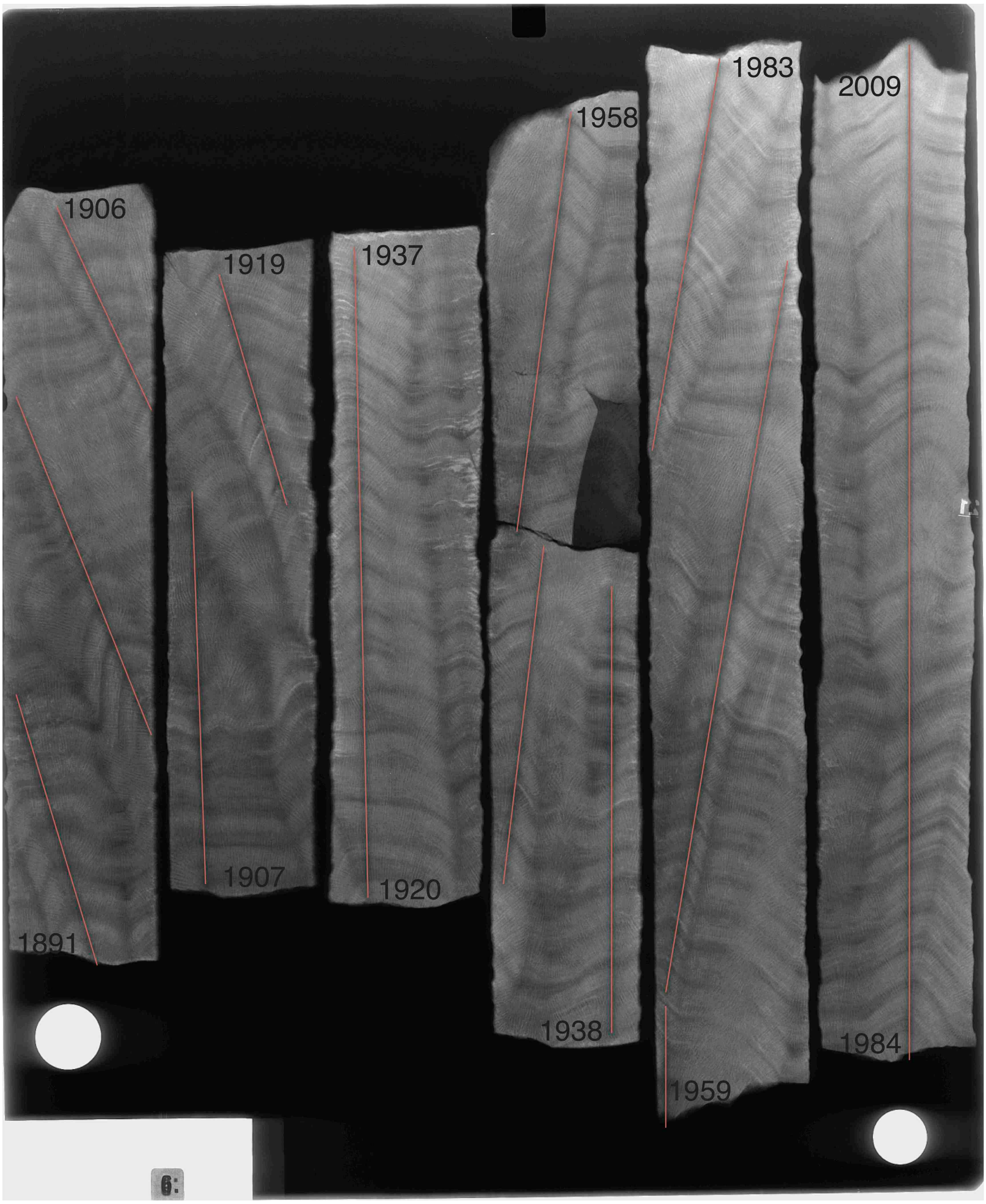

Supplementary Figure 13 - X-ray negative of coral core MER09 slabs from the Mermaid Reef (Rowley Shoals) with sampling transects indicated (red lines).

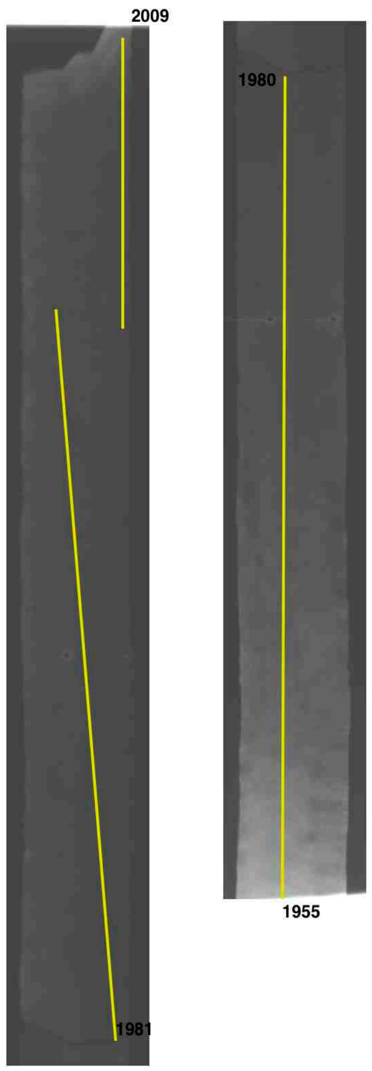

Supplementary Figure 14 - X-ray positive prints of coral core CLE09 slabs from the Clerke Reef (Rowley Shoals) with sampling transects indicated (yellow lines).

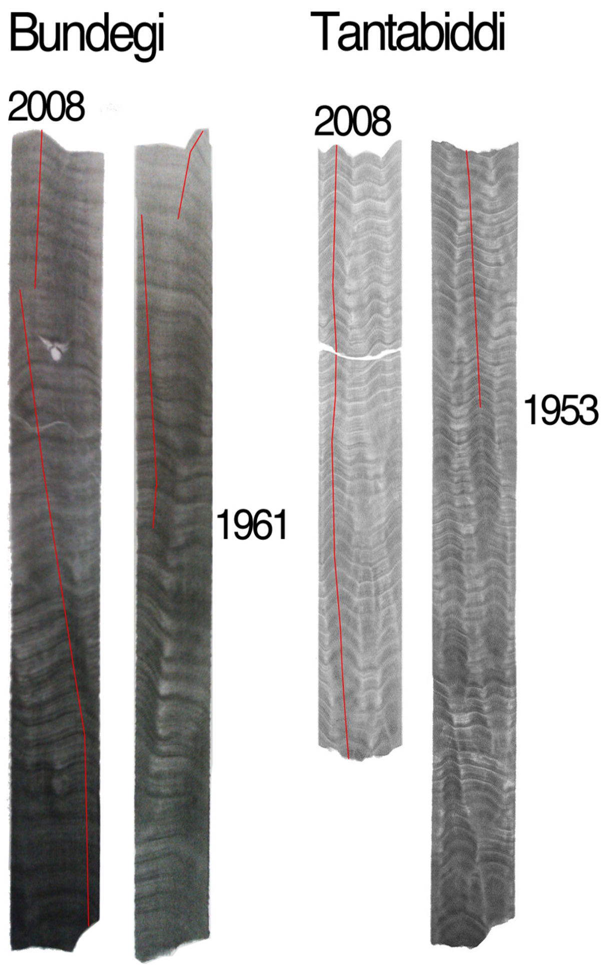

Supplementary Figure 15 - X-ray positive prints of coral core slabs from the Ningaloo Reef (Bundegi (BUN05C) and Tantabiddi Reef (TNT07C)) with sampling transects indicated (red lines).

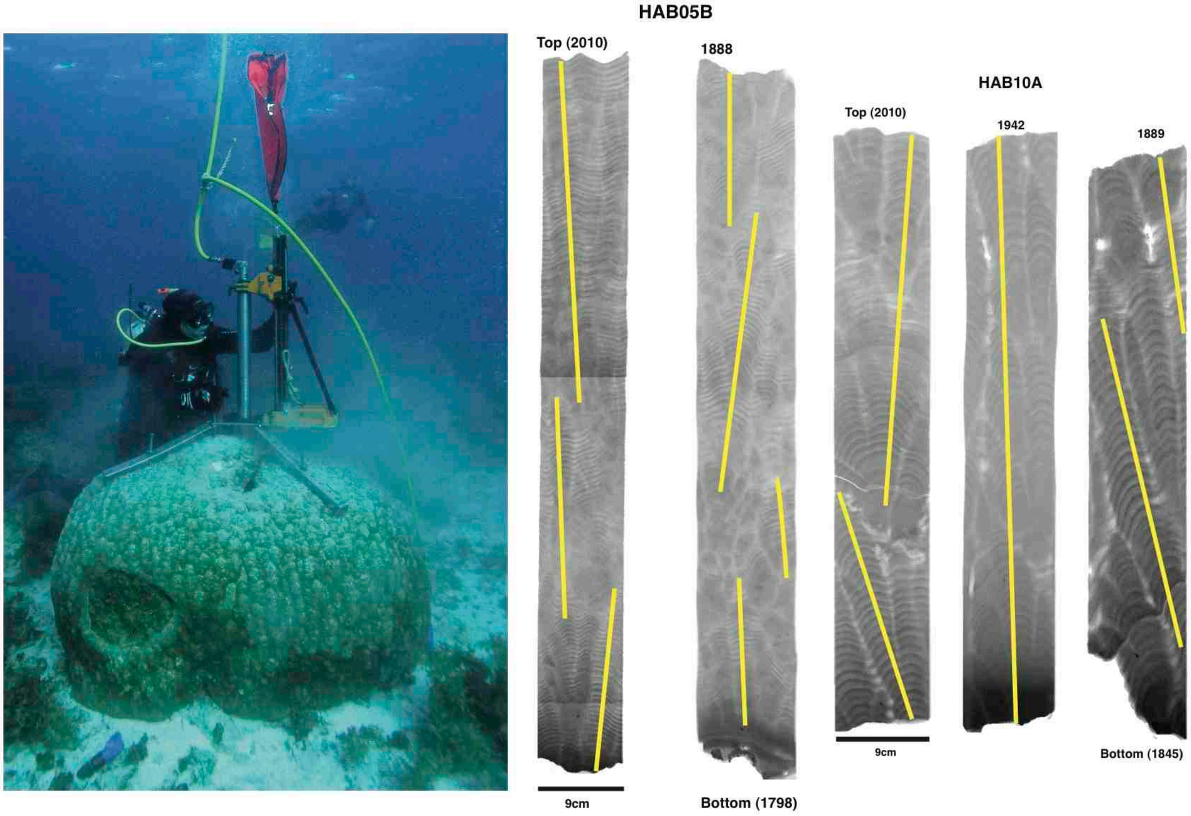

Supplementary Figure 16 – Left) Coring of coral core HAB10A (Photo from E. Matson, AIMS), Right) X-ray positive prints of coral core slabs (HAB05B, HAB10A) from the Houtman Abrolhos Islands (after Zinke et al, 2014[13]) with sampling transects indicated (yellow lines).

	WA comp	RS	NING	HAI	WA ERSST	WA Had1	WA Had3	WA OISST
WA comp	1							
RS	0.91***	1						
NING	0.91***	0.70***	1					
HAI	0.88***	0.65***	0.7***	1				
WA ERSST	0.50***	0.51***	0.32*	0.36**	1			
WA Had1	0.54***	0.50***	0.39*	0.45***	0.77***	1		
WA Had3	0.51**	0.46*	0.43*	0.37**	0.84***	0.82***	1	
WA OISST	0.63***	0.51**	0.67***	0.62***	0.94***	0.92***	0.86***	1

p-values *0.1, **0.05, ***0.001

Supplementary Table 1 – Least squares linear regression between WA coral SST (WA comp), Rowley Shoals (RS), Ningaloo Reef (NING) and Houtman Abrolhos (HAI) mean annual coral SST and 2x2° gridded ERSST[1] (from 1854), 1x1° gridded HadISST[6] (Had1, from 1870), 5x5° gridded HadSST3[14] (Had3, from 1850) and 1x1° gridded OI SST[15] (from 1981), all centered on 17.5°S, 118.5°E. Maximum number of years used for correlations with each SST dataset (see above in brackets).

	WA comp	RS	NING	HAI	WA ERSST	WA Had1	WA Had3	WA OISST
WA comp	1							
RS	0.80***	1						
NING	0.75***	0.31*	1					
HAI	0.60*	0.10	0.25**	1				
WA ERSST	0.37**	0.39***	0.24*	0.15	1			
WA Had1	0.35**	0.32**	0.17*	0.21	0.73***	1		
WA Had3	0.35**	0.28	0.32*	0.19	0.81***	0.80***	1	
WA OISST	0.64***	0.54***	0.66***	0.63***	0.94***	0.94***	0.85***	1

p-values *0.1, **0.05, ***0.001

Supplementary Table 2 – Least squares linear regression between detrended WA coral SST (WA comp), Rowley Shoals (RS), Ningaloo Reef (NING) and Houtman Abrolhos (HAI) mean annual coral SST and 2x2° gridded ERSST[1] (from 1854), 1x1° gridded HadISST[6] (Had1, from 1870), 5x5° gridded HadSST3[14] (Had3, from 1850) and 1x1° gridded OI SST[15] (from 1981), all centered on 17.5°S, 118.5°E. Maximum number of years used for correlations with each SST dataset (see above in brackets).

	WA comp	RS	NING	HAI
ERSST	0.50***	0.43***	0.29*	0.63***
Had1	0.54***	0.48***	0.50***	0.56***
Had3	0.51**	0.43**	0.59***	0.66***
OISST	0.63***	0.53***	0.71***	0.66***

p-values *0.1, **0.05, ***0.001

Supplementary Table 3 – Least squares linear regression between WA coral SST (WA comp), Rowley Shoals (RS), Ningaloo Reef (NING) and Houtman Abrolhos (HAI) mean annual coral SST with site-specific 2x2° gridded ERSST[1] (from 1854), 1x1° gridded HadISST[6] (from 1870), 5x5° gridded HadSST3[14] (from 1850) and 1x1° gridded OI SST[15] (from 1981). Maximum number of years used for correlations with each SST dataset (see above in brackets) and including long-term trend.

	WA comp	RS	NING	HAI
ERSST	0.37**	0.37***	0.26*	0.43***
Had1	0.35**	0.35***	0.29**	0.33**
Had3	0.35**	0.23*	0.42**	0.45***
OISST	0.64***	0.43**	0.70***	0.68***

p-values *0.1, **0.05, ***0.001

Supplementary Table 4 – Least squares linear regression between detrended WA coral SST (WA comp), Rowley Shoals (RS), Ningaloo Reef (NING) and Houtman Abrolhos (HAI) mean annual coral SST with site-specific 2x2° gridded ERSST[1] (from 1854), 1x1° gridded HadISST[6] (from 1870), 5x5° gridded HadSST3[14] (from 1850) and 1x1° gridded OI SST[15] (from 1981). Maximum number of years used for correlations with each SST dataset (see above in brackets).

	IWP06	WA ERSST	WA comp	RS	NING	HAI
**WPG**	-0.50***	-0.22**	-0.39***	-0.17	-0.33**	-0.32**
**WP SST**	0.48***	0.28***	0.38**	0.20*	0.25*	0.30**
**Paleo-WPG**	-0.80***	-0.20*	-0.46***	-0.38***	-0.41**	-0.20*
**IWP06#**	1	0.22**	0.46***	0.35**	0.33**	0.24**
**Niño4**	-0.58***	-0.27**	-0.37***	-0.17	-0.29**	-0.32**

p-values *0.1, **0.05, ***0.001

Supplementary Table 5 – Least squares linear regression between detrended IWP06[24] SST (#1795 to 1992), WA coral SST (WA comp), WA ERSST[1], Rowley Shoals (RS), Ningaloo Reef (NING) and Houtman Abrolhos (HAI) mean annual coral SST with the Western Pacific SST gradient[2] (WPG; since 1854), Western Pacific SST[1] (since 1854), Paleo-WPG and the Niño4 index[3], all between 1854 to 2010 or for maximum years of overlap. Note that correlation of WA coral SST with the Paleo-WPG are higher than those with instrumental WPG and Niño4 indices.

	WA ERSST	WA comp	WA SSH	RS	NING	HAI
**WPG**	-0.48**	-0.69**	-0.84***	-0.41*	-0.73***	-0.45**
**WP SST**	0.51**	0.63*	0.75***	0.37*	0.79***	0.46*
**Niño4**	-0.66***	-0.67***	-0.84***	-0.40	-0.69***	-0.45**

p-values *0.1, **0.05, ***0.001

Supplementary Table 6 – Least squares linear regression between detrended WA ERSST[1], WA coral SST (WA comp), WA sea surface height (SSH) from SODA reanalysis[10], Rowley Shoals (RS), Ningaloo Reef (NING) and Houtman Abrolhos (HAI) mean annual coral SST with the Western Pacific SST gradient[2] (WPG), Western Pacific SST[1] and the Niño4 index[3] between 1980 to 2010.

	WA ERSST	WA comp	WA SSH
**WA ERSST**	1		
**WA comp**	0.69***	1	
**WA SSH**	0.66***	0.70***	1
**WPG**	-0.48**	-0.69**	-0.84***
**WP SST**	0.51**	0.63*	0.75***
**Niño4**	-0.66***	-0.67***	-0.84***

p-values *0.1, **0.05, ***0.001

Supplementary Table 7 – Least squares linear regression between detrended mean annual WA ERSST[1], WA coral SST (WA comp) and WA sea surface height (SSH) from SODA reanalysis[10] with the Western Pacific SST gradient[2] (WPG), Western Pacific SST[1] and the Niño4 index[3] between 1980 to 2010.

Year	JF ERSST	JFM ERSST	Annual ERSST	WA coral SST
1951	-0.48	-0.63	-0.94	-0.27
1952	-1.05	-0.96	-0.46	0.01
1953	-1.10	-0.84	-0.52	-0.08
1954	-0.29	-0.20	-0.20	0.04
1955	0.69	0.57	0.40	0.15
1961	0.99	0.98	0.42	-0.17
1962	0.88	0.68	0.39	0.14
1963	0.38	0.68	0.51	0.21
1967	0.50	0.28	0.32	0.03
1973	0.41	0.29	0.41	0.18
1976	-0.14	-0.10	-0.46	0.22
1977	-0.52	-0.65	-0.30	-0.17
1980	0.56	0.46	0.29	0.01
1983	0.41	0.29	0.20	-0.01
1986	-0.28	-0.15	-0.59	-0.30
1987	-0.94	-0.79	-0.40	0.04
1991	-0.39	-0.34	0.01	0.14
1992	-0.79	-0.78	-0.32	0.01
1993	-0.94	-1.15	-0.61	-0.33
1996	-0.21	-0.07	0.19	0.37
1999	0.64	0.65	0.67	0.67
2003	-0.02	-0.03	-0.34	-0.08
2004	-1.04	-0.93	-0.54	-0.36
2005	-0.46	-0.31	-0.32	-0.41
2006	-0.73	-0.47	-0.36	-0.06
2008	0.47	0.49	0.25	0.51

Supplementary Table 8 – ERSSTv3b[1] anomalies between 1950 to 2010 for the grid box 17-28°S, 113-119°E for warm **Ningaloo Niño (bold red)** and cold **Ningaloo Niña (bold blue)** events as defined in Kataoka et al. (2013)[16]. Note that Ningaloo Niño/Niña was defined for a region (22-28°S, 108-114°E) south of Ningaloo Reef (22°S) to Houtman Abrolhos (28°S) only. SST anomalies were computed for January-February (JF) SST, January to March (JFM) SST and annual average SST between January and December. Last column shows WA coral SST annual anomalies (events not recorded or of different sign are not coloured). SST anomalies have been linearly detrended. The annual mean ERSST captures the warm and cold events similar to the January-February or January to March ERSST, although in general with lower magnitude (recorded events coloured). . JF and JFM ERSST are highly correlated with Annual ERSST (r^2= 0.78, p<0.001; r^2= 0.78, p<0.001, respectively). WA coral SST records fewer events. Annual ERSST and WA coral SST are correlated at r^2= 0.45, p=0.0005, while JFM ERSST is correlated at r^2= 0.28, p=0.009.

	WA ERSST	WA comp	WA SSH	RS	NING	HAI
**NN index**	0.68***	0.59***	0.59***	0.33***	0.51***	0.43***

p-values *0.1, **0.05, ***0.001

Supplementary Table 9 – Least squares linear regression between detrended mean annual WA ERSST[1], WA coral SST (WA comp), WA sea surface height (SSH) from SODA reanalysis[10], Rowley Shoals (RS), Ningaloo Reef (NING) and Houtman Abrolhos (HAI) mean annual coral SST with Ningaloo Niño (January-February) index[16,17] (NN) between 1948 to 2010.

Supplementary References

1. Smith, T.M., R.W. Reynolds, T.C. Peterson, J. Lawrimore, Improvements to NOAA's historical merged land–ocean surface temperature analysis (1880–2006), *J. of Climate* **21**, 2283 (2008).
2. Hoell, A., Funk, C. The ENSO-related West Pacific sea surface temperature gradient. *J. of Climate* **26**, 9545-9562 (2013).
3. Kaplan, A. *et al.* Analyses of global sea surface temperature 1856-1991. *J. Geophys. Res.* **103**, 18567-18589 (1998).
4. Kuhnert, H. *et al.* A 200-year coral stable oxygen isotope record from a high-latitude reef off Western Australia. *Coral Reefs* **18**, 1-12 (1999).
5. Kuhnert, H., Paetzold, J., Wyrwoll, K.H., Wefer, G. Monitoring climate variability over the past 116 years in coral oxygen isotopes from Ningaloo Reef, Western Australia. *International Journal of Earth Sciences* **88**, 725-732 (2000).
6. Rayner, N. A. (2003), Global analyses of sea surface temperature, sea ice, and night marine air temperature since the late nineteenth century. *Journal of Geophysical Research* **108** (D14).
7. Woodruff, S.D. *et al.* ICOADS Release 2.5: Extensions and enhancements to the surface marine meteorological archive. *Int. J. Climatol.*, **31**, 951-967 (2011).
8. D'Arrigo, R. *et al.* The reconstructed Indonesian warm pool sea surface temperatures from tree rings and corals: Linkages to Asian monsoon drought and El Nin˜o–Southern Oscillation. *Paleoceanography* **21**, PA3005, doi:10.1029/2005PA001256 (2006).
9. Trouet, V. and van Oldenborgh, G.J. KNMI Climate Explorer: a web-based research tool for high-resolution paleoclimatology. *Tree Ring Research* **69**, 1, 3-13 (2013).
10. Carton, J. A. & Giese, B. S. A Reanalysis of Ocean Climate Using Simple Ocean Data Assimilation (SODA). *Mon. Wea. Rev.* **136**, 2999–3017 (2008).

11. Wilson, R. *et al.* Reconstructing ENSO: the influence of method, proxy data, climate forcing and teleconnections. *Journal of Quaternary Science* **25**, 62-78 (2010).
12. Emile-Geay, J., Cobb, K. M., Mann, M. E. and Wittenberg, A. T. Estimating Central Equatorial Pacific SST Variability over the Past Millennium. Part II: Reconstructions and Implications. *J. of Climate* **26**, 2329-2352 (2013).
13. Zinke, J. *et al.* Corals record long-term Leeuwin Current variability during Ningaloo Niño/Niña since 1795, *Nature Communications* **5**, 3607, doi:10.1038/ncomms4607 (2014).
14. Kennedy *et al.* Reassessing biases and other uncertainties in sea-surface temperature observations since 1850 part 2: biases and homogenisation. *J. Geophys. Res*. **116**, D14104, doi:10.1029/2010JD015220 (2011).
15. Reynolds, R. W. *et al.* Daily High-resolution Blended Analyses for sea surface temperature. *J. Climate* **20**, 5473-5496 (2007).
16. Kataoka, T., Tozuka, T., Behera, S. and Yamagata, T. On the Ningaloo Niño/Niña. *Clim. Dyn*. **43**, 1463-1482 (2013).
17. Marshall, A.G., Hendon, H.H., Feng, M., Schiller, A. Initiation and amplification of the Ningaloo Niño. *Climate Dynamics* (Online First), doi:10.1007/s00382-015-2477-5 (2015).
